# Particle dissolution rate controls macrophage response and drug release from mesoporous silica inhalation carriers

**DOI:** 10.1038/s41598-026-46033-8

**Published:** 2026-04-01

**Authors:** Tetiana Yalovenko, Jesús Enrique Campos Pacheco, Gustav Sedelius, Emilie Schousboe, Antonia Alionte, Georgia Pilkington, Anna Gustafsson, Sabrina Valetti

**Affiliations:** 1 Biomedical Science, Faculty of Health and Society, Malmö University, Malmö, 205 06 Sweden; 2 Biofilms – Research Center for Biointerfaces (BRCB), Malmö University, Malmö, 205 06 Sweden; 3 Surface and Corrosion Science, KTH Royal Institute of Technology, Stockholm, SE-100 44 Sweden

**Keywords:** Pulmonary drug delivery, Human macrophages, Respiratory infections, Micro/nanocarrier, Amorphous mesoporous silica particles, Clofazimine, Drug discovery, Nanoscience and technology

## Abstract

Mesoporous silica particles (MSPs) are promising micronized carriers for pulmonary drug delivery, combining excellent aerodynamic properties with the ability to dissolve into nanoparticles in lung fluid, enabling intracellular drug transport. In this study, we evaluated the interactions between three MSPs with distinct dissolution profiles in lung fluid and human macrophage models (primary M1, M2, and dTHP-1 cells). Following 4-hour exposure, all MSPs maintained high mitochondrial activity above the IC_50_ threshold, regardless of concentration, up to 1 mg/mL. MSPs did not induce TNF-α release, indicating a minimal immunological response. However, in simulated lung fluid, MSPs dissolved into nanoparticles exhibited greater toxicity than their microparticle counterparts. Toxicity correlated with dissolution rate: faster-dissolving particles were more toxic at short exposure times, while all MSPs reached similar toxicity levels after 24 h, independent of medium composition. Albumin, the predominant lung fluid protein, was found to mitigate particle toxicity, enhancing biocompatibility. Drug release studies revealed that faster-dissolving particles produced a rapid but unstable supersaturated drug solution, whereas slower-dissolving particles offered a more sustained release profile, supporting its potential for prolonged pulmonary therapy. The lowest observed adverse effect level (LOAEL) *in vitro* was determined to be 0.06 mg/mL for all the MSPs. This concentration maintained mitochondrial activity, oxidative stress and cell membrane integrity above 70%. These findings highlight the translational potential of MSPs as dual-scale carriers for targeted pulmonary delivery, combining effective drug release with favorable safety profiles.

**Supplementary Information:**

The online version contains supplementary material available at 10.1038/s41598-026-46033-8.

## Introduction

The delivery of drug agents via the pulmonary route is an area of extensive research^1^. Compared with other routes, such as oral and parenteral routes, pulmonary drug delivery offers several advantages, such as the possibility of both site-directed and systemic delivery, faster onset of action, high bioavailability, circumvention of first-pass metabolism and, importantly, fewer systemic side effects^2^. Recently, the utilization of nano- and microparticles as drug carriers in targeted therapy has garnered considerable attention^3^. The formulation of systems incorporating drug molecules within a carrier, whether embedded in the matrix or absorbed onto the surface, represents a new stage toward achieving enhanced therapeutic outcomes^4^. This is driven by the potential to increase drug deposition in airways.

Despite significant progress in the field of pulmonary drug delivery, the search for an “ideal” therapeutic carrier is still ongoing. The “ideal” drug carrier should fulfill and combine biocompatibility, targeting ability, controlled drug release and stability. Among inhaled formulations, dry powders are attractive due to patient compliance, room-temperature stability, and simplified storage and distribution, which can help mitigate logistical challenges in global healthcare. Mesoporous silica particles (MSPs) show considerable promise as drug carriers for dry powder formulations because of their high surface area, tunability and mesoscale pores make them suitable for encapsulating small drugs and biopharmaceuticals. In a previous study, our research group demonstrated successful clofazimine (CLZ) loading into MSPs, formulating a dry powder formulation with a high respirable fine particle fraction (FPF = 50%) and a drastic increase in the apparent solubility of the drug (16-fold greater than that of the pure drug) in simulated lung fluid. This strategy offered a dual micro/nano approach for extra and intracellular delivery, reducing the mycobacterial burden inside macrophages, increasing the drug concentration in a Calu-3 respiratory epithelium model^5^ and demonstrating no measurable toxicity of the carrier with macrophages and Calu-3 cells^6^. Moreover, MSPs have been demonstrated to be suitable as the sole excipient for delivery into the lungs of other small molecules, such as budesonide^7^ and proteins, retaining enzymatic activity in the lung fluid^6^. MSPs are already “generally recognized as safe” (GRAS) by the US Food and Drug Administration (FDA) for topical administration^8^. Importantly, MSPs are composed solely of amorphous silica, which differs significantly from crystalline silica, which is known for its association with silicosis, particularly in terms of its safety profile and solubility in biological fluids^5,8,9^. However, a more detailed and comprehensive evaluation of how amorphous MSPs interact with lung tissue is needed before they can be considered for clinical practice. Any micro- or nanoparticles that intentionally or unintentionally enter airways are in contact with phagocytic cells of the immune system. Alveolar macrophages (AMs), which predominate the alveoli of healthy individuals, are the most important cells of innate immunity and are actively involved in various pulmonary diseases. AMs clear inhaled particulates and constitute the first-line defense against foreign antigens and pathogens. Depending on various properties of the carrier, including the chemical composition, size, surface area, and type of targeted cells, they can potentially demonstrate toxicity toward cells and induce inflammatory responses^10,11^. Therefore, the natural ability of monocytes and macrophages to easily phagocytose foreign material is an important research focus and potential endpoint for drugs encapsulated in micro- and nanoparticles, such as MSPs^9,12,13^. The high ability of AMs to internalize foreign particles (including MSPs as drug carriers) could be a “Trojan horse” strategy to deliver elevated drug concentrations to cells, which are pivotal in the immune system’s response to specific diseases, such as tuberculosis^5^.

AMs are generally divided into two subsets (M1 and M2), although phenotypes are distributed along a spectrum and rarely fit this binary classification, as many phenotypes exist between the two extremes^14^. AMs change their phenotype in response to various environmental factors toward the classically activated M1 type (proinflammatory, mediating resistance to pathogens) or the alternatively activated M2 type (anti-inflammatory, promoting tissue remodeling)^15^. The M2 types are further divided into three subsets, M2a, M2b and M2c^14^. AMs are thought to be mainly of the M2c subtype in a healthy state, thereby preventing unwarranted inflammatory responses to harmless materials encountered in the lung. In contrast, during pathogen invasion, AMs polarize into the classically activated M1 phenotype due to elevated levels of proinflammatory cytokines, such as interferon gamma (IFN-γ), combined with the triggering of pattern recognition receptor activation. The M1/M2 polarization states represent the endpoints of a broad, continuous functional and phenotypic spectrum, illustrating the plasticity and heterogeneity inherent in human macrophages^16^. Gallud et al. investigated the influence of macrophage polarization status (M1 and M2) on two MSPs of micron (diameter = 2270 ± 200 nm) and submicron (diameter = 200 ± 50 nm) size^9^. They reported, in agreement with Kusaka and colleagues^17^, that the submicron particles induced a greater inflammatory response than did the micron-sized particles.

From the perspective of pulmonary drug delivery, the dissolution of MSPs could also play a crucial role in macrophage inflammatory responses. Braun et al. reported that the dissolution behavior of amorphous silica particles in various biological fluids, including lung fluid, is significantly influenced by their surface area^18^. In agreement with this report, a recent study by some of the present authors revealed a direct proportionality between the surface area of the particles, the *in vitro* dissolution rate and the in vivo clearance of the particles from the lungs of the rats^19^. We have also previously shown that microsized MSPs spontaneously dissolve in lung fluid, creating nanoparticles via the so-called dual micro-nano approach^5,6^. In this context, this novel approach refers to the use of inhalable micron-sized carriers that provide optimal aerodynamic deposition in the lower airways and subsequently dissolve in lung fluid to generate nanoparticles in situ*in situ*, thereby enabling enhanced intracellular delivery. This micro-nano strategy is fundamentally distinct from conventional microparticle formulations, which struggle to target or penetrate the infected tissues once deposited in the lungs.

Here the aim of this study was to evaluate the impact of this dual micro/nano approach on human macrophage-like cells. Since primary cells obtained from different donors may display variability in cell behavior and cell lines may not fully recapitulate the complexity and heterogeneity of in vivo*in vivo* conditions, we combined different *in vitro* models in experiments to obtain results closer to the in vivo*in vivo* situation. As the dissolution profile of the carrier is important, we tested three different MSPs characterized by different half-time dissolution rates (T50%) in the SLF. The dissolution kinetics of particles produced with comparable synthesis methods and surface properties have been quantified previously, providing a relevant reference framework for the expected behavior of our MSPs panel^19^. The cytotoxic effects of the particles were investigated by evaluating mitochondrial activity, reactive oxygen species (ROS) levels and lactate dehydrogenase (LDH) levels. The release of TNF-α was measured to understand the inflammatory response. Since proteins present in airways can contact the surface of particles and therefore influence cellular responses, the cytotoxic effects of MSPs dissolved in SLF in the presence of proteins were also investigated. While findings from cell culture models may not directly translate to the in vivo*in vivo* conditions of an individual patient, evaluating nanocarriers in these models is crucial for minimizing the potential for adverse reactions or toxic effects. Finally, the impact of different properties of MSPs (i.e., dissolving properties, pore volume, surface area) on the *in vitro* drug dissolution of a selected lung therapeutic, CLZ, was assessed as a case study by determining if the released drug reached a therapeutic concentration.

## Materials and methods

### Materials

All amorphous MSPs were obtained from Nanologica A.B. (Södertälje, Sweden). Filters with sizes of 13 mm and 25 mm and pore sizes of 0.2 μm polytetrafluoroethylene (PTFE) were purchased from VWR (Stockholm, Sweden). Magnesium chloride hexahydrate, sodium chloride, potassium chloride, sodium sulfate, sodium acetate trihydrate, sodium citrate dihydrate and sodium hydrogen carbonate were obtained from Sigma‒Aldrich (Tyreso, Sweden). Calcium chloride dihydrate and lipopolysaccharide (LPS) from *Escherichia coli* serotype 0111:B4 were obtained from Merck (Solna, Sweden). RPMI 1640 medium (ATCC modification), RPMI 1640 medium without phenol red, FBS, penicillin/streptomycin solution, phosphate-buffered saline (PBS), Dulbecco’s phosphate-buffered saline (DPBS) supplemented with calcium and magnesium, granulocyte‒macrophage colony-stimulating factor (GM-CSF), macrophage colony-stimulating factor (M-CSF), human IFN‒gamma recombinant protein (IFN‒𝛾), human interleukin 10 (IL‒10), Hanks’ balanced salt solution (HBSS) and Accutase were purchased from Gibco™, Fisher Scientific GTF AB (Gothenburg, Sweden). Phorbol 12-myristate 13-acetate (PMA), 2-(3,5-diphenyltetrazol-2-ium-2-yl)−4,5-dimethyl-1,3-thiazole, and bromide (MTT) were purchased from Fisher Scientific GTF AB (Gothenburg, Sweden). 7-AAD, LDH Cytotoxicity Assay, CyQUANT™, H2DCFDA (H2-DCF, DCF) molecular probes and Acridine Orange (10 mg/mL in water) were obtained from Invitrogen™, Fisher Scientific GTF AB (Gothenburg, Sweden). Bovine serum albumin (BSA) and Triton X-100 were purchased from Sigma‒Aldrich (Tyreso, Sweden). Human TNF-𝛼, human IL-10, human CCL2/MCP-1, and DuoSet ELISAs were obtained from BioTechne Ireland Limited (Dublin, Ireland). Dimethyl sulfoxide (DMSO) was purchased from Fisher BioReagents™, Fisher Scientific GTF AB (Gothenburg, Sweden). Ficoll-Paque PLUS was obtained from GE Healthcare (Uppsala, Sweden). Monocyte attachment medium (MAM) was obtained from PromoCell, Mediqip (Älvsjö, Sweden). Antibodies against CD80-APC (Clone REA661) and CD206-PE (Clone DSN228) were obtained from Miltenyi Biotec Norden AB (Lund, Sweden). The antitubercular drug clofazimine (CLZ) was purchased from Chemtronica A.B. (Stockholm, Sweden). The lung lipid surfactant dipalmitoylphosphatidylcholine (16:0 PC, DPPC) was purchased from Avanti^®^ (New York, USA).

### Mesoporous silica particles

All the amorphous MSPs studied, referred to here as MSP-I, MSP-II and MSP-III (Table 1), were provided by Nanologica A.B., Södertälje, Sweden. MSP-I corresponds to the commercially available silica SUNSPHERE^®^ H-32 from Asahi Glass (AGC Group) after size classification to achieve a mean particle size and polydispersity (d90/d10) suitable for inhalation, as reported elsewhere^19^ with the name CAP-558. MSP-II and MSP-III were synthesized by Nanologica AB using the sol-gel method by Nanologica AB (publ) using their proprietary method previously published^20^. Briefly, the sol-gel process consisted in the spraying of a silica precursor to produce spherical particles with a reasonably narrow particle size range 5–20 microns. The porosity arises in these particles during the condensation and drying of the silica gel which yield a broader pore size compared with the templated synthesis route. The surface area, pore volume and pore size are strongly influenced by condensation conditions and can be easily varied in order to produce a variety of particles with differing textual properties. The results of dry powder formulations prepared with MSP-III have already been reported with the general names of MSPs^5,6^ or NAP-304^19^.

### Nomenclature of macrophage differentiation, activation, and polarization

The description of macrophage differentiation, activation and polarization is currently contentious and confusing. We used the macrophage nomenclature described by Murray et al.^21^. The monocyte cell line THP-1 is differentiated into macrophage-like cells (herein named dTHP-1) via PMA. Human peripheral blood mononuclear cells (PBMCs) are differentiated into classically activated M1 type (M1) or alternatively activated M2 type (M2) with GM-CSF or M-CSF, respectively. We use the term ‘‘activation’’ to refer to the perturbation of macrophages with exogenous agents in the same vein, as many use ‘‘polarization.’ Fully active or polarized M1 macrophages are further obtained after stimulation with IFN-γ and LPS, herein named M1 (IFN-γ/LPS). Fully active or polarized M2 macrophages are further obtained after stimulation withIL-10, herein named M2 (IL-10).

**Table 1:** Individual properties of the MSPs used in this work.

Property, Unit	MSP-I	MSP-II	MSP-III
*Mean particle size*,* µm*	2.2	2.2	2.2
*Pore volume, cm*^3^/g	1.63	1.00	0.70
*Surface area, m*^2^/g	511	380	312
*Pore size*,* Å*	128	105	90
*Dissolution in SLF (t50%)*, h*	2	4	6

* Rounded to the nearest hour^19^.

Physicochemical parameters (pore volume, surface area, pore size, and dissolution half-time) were provided by the manufacturer (Nanologica AB) as representative product specifications.

### THP-1 cell line culture

The THP-1 (TIB-202) cell line was purchased from American Type Culture Collection (ATCC, Manassas, VA, USA). The cells were used at passages 4–20 and were grown at 37 °C and 5% CO_2_. THP-1 cells are monocytes isolated from the peripheral blood of acute monocytic leukemia patients. The ATCC guidelines for thawing and maintaining cells were strictly followed. The cell culture medium was RPMI 1640 (ATCC modification) supplemented with 20% (for the first 3 passages after thawing) and 10% (for all the next passages) heat-inactivated FBS and 1% penicillin/streptomycin (10,000 units/mL penicillin; 10,000 µg/mL streptomycin). A trypan blue exclusion test was performed regularly to assess viability and cell counts. Media feeding occurred every 3–4 days, with cell passage performed every 7 days.

To induce the differentiation of THP-1 cells into macrophage-like cells, 4 × 10^4^ cells per well were incubated in 96-well plates (Sarstedt, Cell+, Flat Base, Yellow, Germany) at 37 °C with culture media supplemented with 50 ng/mL PMA for 48 h. Subsequently, the cells were washed thoroughly with 200 µL per well with prewarmed (37 °C) PBS to obtain adherent PMA-differentiated THP-1 cells (dTHP-1).

### Differentiation of human monocyte-derived macrophages

Leukocyte concentrates from healthy donors were purchased from the local blood donation bank (Skåne University Hospital, SUS, Lund, Sweden) and separated by density centrifugation via Ficoll‒Paque PLUS. Monocytes were enriched based on adherence and differentiated into M1‑ or M2‑like macrophages using GM‑CSF or M‑CSF, respectively. M1 activation was induced with IFN‑γ and LPS, while M2 activation was achieved with IL‑10. Supernatants were collected for cytokine analysis, and cells were harvested for flow cytometry as described in Sect. “Flow cytometry”, "Quantitative analysis of TNF-*a*, IL-10 and CCL2", S2 and S3. The PBMC protocol^22,23^ was performed in triplicate with cells from three different donors to ensure reproducibility. A detailed protocol for PBMC isolation, macrophage differentiation and activation is provided in the Supplementary Information (Method S1).

### Flow cytometry

To evaluate the phenotype after THP-1 differentiation and human primary macrophage polarization, the surface markers CD80 and CD206 were measured via flow cytometry (BD Accuri™ C6 Plus, Biosciences, Le Pont de Claix, France) and BD Accuri C6 Plus Software, version 1.0.34.1. A total of 10,000 events were collected at the defined gates. Detailed description of the experimental procedure is provided in the Supplementary Information (Method S2).

### Simulated lung fluid (SLF)

SLF was prepared freshly prepared in accordance with the method described in the literature as SLF1^24^. The compositions of the salts are tabulated in the Supplementary Information, Table S1.

### Cytotoxicity study of MSPs

The cytotoxicity of MSPs on macrophages was investigated by evaluating mitochondrial activity via the MTT test. This colorimetric assay assesses cell viability on the basis of the activity of cytoplasmic NADPH-dependent oxidoreductases and relies on the capacity of viable cells to convert 3-^25^−2,5 diphenyltetrazolium bromide (MTT) (yellow tetrazolium salt), which is an indicator of redox enzyme function, into formazan through mitochondrial succinate dehydrogenase activity^24,26^. dTHP-1, M1 and M2 cells were incubated in 96-well plates with eight different concentrations of MSP-I, MSP-II, and MSP-III suspended in PBS or SLF, ranging from 0.008 mg/mL to 1 mg/mL, at 37 °C and 5% CO2 for 4 h, 8 h, 12 h and 24 h. When MSPs were suspended in PBS, 50 µL of fresh cell culture medium (i.e., 10% FBS) was added into the well together with 50 µL of MSPs (i.e., final FBS concentration of 5%). The presence of proteins from the serum influences the interaction between MSPs and cells^9,27^. Likewise, albumin, a major component of lung fluids, has been reported to affect particle phagocytosis in dTHP-1 cells^28^. To investigate the influence of proteins in the medium, dTHP-1 cells were incubated for 4 h with 0.06 or 0.5 mg/mL MSPs suspended in SLF supplemented with 1:1 medium (i.e., final FBS concentration of 5%) or 8.8 mg/mL BSA. Visual inspection and gentle agitation confirmed the absence of macroscopic sedimentation during the exposure period. For all the conditions tested, after the appropriate incubation time, the cells were washed thoroughly with 200 µL of preequilibrated (37 °C) PBS per well. Next, 100 µL of new PBS and 10 µL of 5 mg/mL MTT reagent in PBS were added to all the wells except the blank well, which was used as an absorbance interference control. Following 2.5 h of incubation, the MTT solution was carefully removed, and 100 µL of DMSO was added to solubilize the formazan crystals. For the blank control wells, 100 µL of PBS was added instead. The plates were placed on a gentle shaker for 10 min of homogenization while being protected from light. The absorbance of each solution was measured at a wavelength of 570 nm via a Tecan Safire microplate reader (Florida, USA). Untreated samples (or negative controls) were prepared by incubating the cells with the same medium and conditions without MSPs. The absorbance interference from the cells was taken into consideration by subtracting the absorbance from the blank well. The percentage of mitochondrial activity was calculated as the ratio of the treated cells to the untreated cells. 2% Triton X-100 in PBS was used as a toxic positive control to induce cell death and ensure the effectiveness of the assay.

### Quantitative analysis of TNF-α, IL-10 and CCL-2

Inflammatory responses of THP‑1–derived and primary human macrophages were assessed by measuring the secretion of TNF‑α, IL‑10, and CCL‑2 using DuoSet ELISA kits (R&D Systems, Minneapolis, MN, USA) according to the manufacturer’s instructions. RPMI 1640 medium served as a negative control, while IFN‑γ (50 ng/mL) combined with LPS (10 ng/mL) for 20 h was used as a positive control for TNF‑α induction. The assay ranges were 15.6–1,000 pg/mL for TNF‑α and CCL‑2, and 31.3–2,000 pg/mL for IL‑10. Detailed ELISA procedures are provided in the Supplementary Information (Method 3).

### Reactive oxygen species (ROS) detection

Intracellular ROS were identified by the oxidation response of the fluorescent probe 2′,7′-dichlorodihydrofluorescein diacetate (H2DCFDA) in strict accordance with the manufacturer’s recommendations. Briefly, THP-1 cells were differentiated into macrophages in two types of 96-well plates: transparent (Sarstedt, Cell+, Flat Base, Yellow, Germany) and black/clear bottom plates (Corning™ 3603), as described in Sect. "THP-1 cell line culture", in phenol red-free medium (RPMI 1640 medium, Gibco™). No significant difference in fluorescence was detected between the abovementioned plates. The oxidative effects of MSP-I, MSP-II and MSP-III were studied at particle concentrations of 0.008, 0.06, 0.125, 0.5 and 1 mg/mL, respectively, in media such as PBS and SLF. After 4 h of incubation, the cells were washed carefully with 200 µL of lukewarm DPBS with calcium and magnesium and stained with 100 µL of 10 µM H2DCFDA in PBS at 37 °C for 45 min. After incubation, the cells were washed twice with 200 µL of lukewarm DPBS and resuspended in 100 µL of PBS. Fluorescence was detected via a Tecan Safire microplate reader with an excitation/emission wavelength of 485/530 nm for the 96-well plates. Untreated cells in the appropriate medium without MSPs were used as negative controls. Unstained untreated cells were used as blanks. H_2_O_2_ (50 µM) incubated for 45 min was used as a positive control. ROS production was calculated as the fluorescence intensity divided by the mitochondrial activity from the MTT assay^29^. The untreated cells were arbitrarily set to 100% to rule out ROS production due to the PMA treatment. Joshi et al. proposed a mechanism by which amorphous silica particles are phagocytosed by macrophages. Phagosomes containing silica particles leak into the cytoplasm, leading to apoptosis, and leakage has been linked to ROS. Accordingly, the incubation time used herein to measure ROS (i.e., 4 h) is linked to mitochondrial dysfunction during apoptosis or inflammation^30^.

### Lactate dehydrogenase (LDH) release assay

An LDH cytotoxicity assay was conducted following the supplier´s instructions. First, the optimum dTHP-1 cell number was determined to be 25 × 10^3^ cells/well. Therefore, 25 × 10^3^ THP-1 cells/well were differentiated into macrophages (i.e., dTHP-1) in 96-well plates, as described in Sect. "THP-1 cell line culture". The dTHP-1 cells were treated with MSP-I, MSP-II, or MSP-III at particle concentrations of 0.008 mg/mL, 0.06 mg/mL, 0.125 mg/mL, or 1 mg/mL suspended in PBS with cell culture medium (i.e., 50 µL/50 µL, final concentration of 5% FBS), or with pure SLF. After 4–24 h of incubation, the supernatants were harvested and then centrifuged at 12,000 × g for 10 min to remove cell debris and MSPs. The supernatants were subsequently aliquoted into new 96-well plates, and 50 µL of the reaction mixture was added to each sample. Each plate was incubated for 30 min at room temperature in the dark before 50 µL of Stop Solution was added to each sample. The absorbance was measured at 490 nm and 680 nm (background) via a Tecan Safire microplate reader (Florida, USA). The % cytotoxicity level expressed as LDH release was estimated via spontaneous LDH activity controls (cells treated with sterile ultrapure water) and maximum LDH activity controls (cells treated with lysis buffer) and calculated via Eq. 1:

1 $$\:\mathrm{\%}\:\mathrm{C}\mathrm{y}\mathrm{t}\mathrm{o}\mathrm{t}\mathrm{o}\mathrm{x}\mathrm{i}\mathrm{c}\mathrm{i}\mathrm{t}\mathrm{y}\:\mathrm{e}\mathrm{x}\mathrm{p}\mathrm{r}\mathrm{e}\mathrm{s}\mathrm{s}\mathrm{e}\mathrm{d}\:\mathrm{a}\mathrm{s}\:\mathrm{L}\mathrm{D}\mathrm{H}\:\mathrm{r}\mathrm{e}\mathrm{l}\mathrm{e}\mathrm{a}\mathrm{s}\mathrm{e}=\frac{\mathrm{M}\mathrm{S}\mathrm{P}\mathrm{s}\:\mathrm{t}\mathrm{r}\mathrm{e}\mathrm{a}\mathrm{t}\mathrm{e}\mathrm{d}\:\mathrm{L}\mathrm{D}\mathrm{H}\:\mathrm{a}\mathrm{c}\mathrm{t}\mathrm{i}\mathrm{v}\mathrm{i}\mathrm{t}\mathrm{y}-\:\mathrm{S}\mathrm{p}\mathrm{o}\mathrm{n}\mathrm{t}\mathrm{a}\mathrm{n}\mathrm{e}\mathrm{o}\mathrm{u}\mathrm{s}\:\mathrm{L}\mathrm{D}\mathrm{H}\:\mathrm{A}\mathrm{c}\mathrm{t}\mathrm{i}\mathrm{v}\mathrm{i}\mathrm{t}\mathrm{y}}{\mathrm{M}\mathrm{a}\mathrm{x}\mathrm{i}\mathrm{m}\mathrm{u}\mathrm{m}\:\mathrm{L}\mathrm{D}\mathrm{H}\:\mathrm{A}\mathrm{c}\mathrm{t}\mathrm{i}\mathrm{v}\mathrm{i}\mathrm{t}\mathrm{y}-\mathrm{S}\mathrm{p}\mathrm{o}\mathrm{n}\mathrm{t}\mathrm{a}\mathrm{n}\mathrm{e}\mathrm{o}\mathrm{u}\mathrm{s}\:\mathrm{L}\mathrm{D}\mathrm{H}\:\mathrm{A}\mathrm{c}\mathrm{t}\mathrm{i}\mathrm{v}\mathrm{i}\mathrm{t}\mathrm{y}}\:\mathrm{x}\:100$$

### Lysosomal membrane permeability assay

Acridine Orange (AO) is a weak base that readily penetrates cellular membranes and accumulates in acidic lysosomal compartments. Upon lysosomal membrane permeabilization (LMP), AO leaks from lysosomes into the cytosol, where it exists at lower concentrations and therefore emits green fluorescence. Because the increase in cytosolic AO directly corresponds to the loss of lysosomal membrane integrity, monitoring the green fluorescence signal provides a sensitive and quantitative readout of LMP under the experimental conditions^31,32^.

dTHP-1 cells were grown in 96-well black/clear bottom plates (Corning™ 3603), as described in Sect. "THP-1 cell line culture" then incubated with MSP-I, MSP-II and MSP-III (0.06, 0.125 and 0.5 mg/ml in PBS each) for 4 h at 37 °C. After incubation PBS with MSPs was removed and 100 µl of AO staining solution/each well (5 µg/ml) was added for 15 min at 37 °C. The cells were then washed with 100 µl of pre-warmed HBSS 2 × 5 min and resuspended in 100 µL of PBS for fluorescence measurement. Fluorescence was detected via a Tecan Safire microplate reader with an excitation/emission wavelength of 485/535 nm for the 96-well plates. The increase in the intensity of the green fluorescent signal compared to the untreated control was evaluated as the release of AO from lysosomes into the cytosol, indicating LMP.

### Preparation and characterization of CLZ-loaded MSPs

Loading of CLZ into MSP-I and MSP-III involved suspending the particles in a drug solution and then evaporating the solvent, as stated in our previous studies^5,12^. In summary, a concentrated solution of CLZ (2 mg/mL) in acetone was gently stirred with each MSP (40 mg/mL) at room temperature for 2 h. To eliminate any residual water in the particles, the MSPs were dried at 120 °C under vacuum for 3 h before loading. The solvent was subsequently removed via rotary evaporation under reduced pressure (250 bar) at room temperature, followed by an additional 2 h step at 40 °C. The resulting CLZ-MSP-I and CLZ-MSP-III, which were prepared with low drug loading (3–4% w/w), were then air-dried overnight.

To determine the amount of drug loaded in the MSPs (i.e., potency), 10 mg of CLZ-MSP-I or CLZ-III was sonicated in 50 mL of methanol for 1 h to dissolve the CLZ. After centrifugation at 3000 × g for 15 min, the supernatant was filtered through 13 mm PTFE filters (0.2 μm pore size), and the drug content was analyzed via HPLC (see Sect. "HPLC method for drug quantification").

### CLZ release from CLZ-MSPs in simulated lung fluid

In triplicate experiments, drug release from CLZ-loaded MSP-I and -III, which contained 3–4% (w/w) drug, was evaluated in simulated lung fluid at a concentration of 30 mg/L following our previous study^5^. The simulated lung fluid, here denoted as SLF + DPPC, is composed of the salts tabulated in the Supplementary Information (Table S1) with the addition of 0.02% dipalmitoylphosphatidylcholine (DPPC), prepared following a reported protocol^33–35^. The experimental conditions involved maintaining the system at 37 °C and 90 rpm via an Aqua Pro shaking water bath. Samples were withdrawn at various intervals over 24 h, with replacement of withdrawn with SLF + DPPC to maintain a constant volume. After withdrawal, the samples were centrifuged to separate any remaining particles from the solubilized drug, which was assumed to remain in the supernatant. The pellets of the samples were washed three times with Milli-Q water to remove excess salts and then dried via a GeneVac EZ-2 Plus SpeedVac (Ipswich, United Kingdom). The supernatant samples were mixed with methanol, filtered through 13 mm PTFE filters (0.2 μm pore size) and then subjected to HPLC analysis (see Sect. "HPLC method for drug quantification"). The percentage of drug released was calculated as the ratio of the dissolved drug to the initial dose of CLZ-MSP-I or CLZ-MSP-III. SEM analysis to observe morphological changes during dissolution was performed in a separate experiment using unloaded MSP-I, MSP-II, and MSP-III exposed to identical SLF conditions (see Sect. "Ex situ analysis of dissolved CLZ-MSPs by SEM").

### HPLC method for drug quantification

The release of CLZ from CLZ-MSP-I and CLZ-MSP-III was quantified via an Agilent 1100 HPLC system (Santa Clara, USA). The data collated during analysis were obtained from the program OpenLAB CDS (Agilent, Santa Clara, USA) via the HPLC method described previously^5^. Briefly, an isocratic method was employed utilizing a 4.6 × 150 mm column (3.5 μm, SVEA C18 column, Nanologica AB, Södertälje, Sweden). Mobile phase A was composed of Milli-Q water with 0.05% v/v trifluoroacetic acid (TFA; VWR, Stockholm, Sweden), while mobile phase B was composed of acetonitrile, which was mixed at a ratio of 40:60. The injection volume was 50 µL, and the flow rate was 0.8 mL/min. The column temperature was maintained at 40 °C, and the detection wavelength was set to 286 nm. Each analysis included a collection time of 7 min.

To determine the potency of CLZ loaded in CLZ-MSP-I and CLZ-MSP-III, a six-point calibration curve was constructed. A stock solution was prepared by suspending 4.8 mg of CLZ in 50 ml of methanol, resulting in a 96 µg/mL stock concentration. The stock solution was then sonicated for 30 min in a Branson 1800 to accelerate the dissolution of the drug. Standard solutions were prepared from the stock solution by performing a series of dilutions in methanol to obtain concentrations within the range of 0.16–5 µg/mL. The standard solutions were then filtered through a 13 mm, 0.2 μm PTFE filter before HPLC analysis.

To determine the concentration and percentage of CLZ released from the silica particles in SLF + DPPC, a calibration curve was constructed following the same method as previously described. When the standard solutions were prepared, a dilution series was instead performed in methanol and SLF 4, mixed at a ratio of 1:1, to achieve concentrations ranging from 0.16 µg/mL–5 µg/mL. The standard solutions were then filtered through a 13 mm, 0.2 μm PTFE filter before HPLC analysis.

### Ex situ analysis of dissolved CLZ-MSPs by SEM

Empty MSPs and after 24 h-dissolution in SLF (see Sect. "CLZ release from CLZ-MSPs in SLF") were mounted onto a conductive support using carbon tape. An automatic sputter coater (Agar Scientific, Stansted Mountfitchet, United Kingdom) was used to apply a gold coating to the samples, enabling conductivity for the investigation of MSP morphology, as described in^5^. The samples were observed at 2500×, 25,000× and 50,000× magnifications via an EVO LS 10 microscope (Zeiss, Oberkochen, Germany). SEM images were obtained with Smart SEM software (Zeiss, Oberkochen, Germany).

### Statistical analysis of drug dissolution

All analyses were performed via Excel (Microsoft Office) and GraphPad Prism v.10.6.1 (GraphPadSoftware Inc., USA). ANOVA and post hoc tests (Tukey´s test) were used to prove any statistical significance in the samples from the cell studies. Dixon’s Q test with 95% confidence was used for identification and rejection of outliers. The mean value of each replicate ± the standard deviation (SD) or ± standard error of the mean (SEM) was calculated to assess the variation in the replicates, technique and instrument (HPLC). To determine if there was a statistically significant difference in the release concentrations of CLZ between the time points, ANOVA and post hoc tests (Holm’s test) were performed in Jamovi (version 2.3 and 2.4.).

## Results and discussion

### The dose‒dependent effects of MSPs on mitochondrial activity and inflammatory responses in different macrophage-like cells

#### Phenotypic study of the obtained human macrophage-like cells

To obtain reliable results, it is very important to use suitable *in vitro* models of AMs, as these can provide powerful tools to gain valuable insights into toxicity, inflammatory responses and clearance of aerosolized pharmaceutical formulations^36^. There are no generally recognized *in vitro* models for human AMs, as they are difficult to retrieve and quickly lose their specific functions in culture^16^; however, different types of macrophage-like cells have been used for research purposes^22,37^. Therefore, to mimic AMs *in vitro*, it is common to differentiate human blood monocytes (i.e., primary cells) into classically activated M1 cells or alternatively activated M2 cells via GM-CSF or M-CSF, respectively^22^. Fully polarized M1 or M2 macrophages are further obtained after stimulation with IFN-γ/LPS or IL-10. However, primary cells may exhibit variability due to differences between donors, whereas cell lines provide a consistent and homogeneous cell population, thus ensuring the reproducibility of experimental results. To overcome this robustness challenge, the THP-1 human leukemia monocytic cell line is a widely accepted cell line for studying macrophage functions, signaling pathways and the transport of nutrients and drugs^37^. THP-1 cells can be differentiated into a macrophage-like phenotype (dTHP-1) via the use of PMA^30^. To profile the phenotype of the macrophage-like cells obtained, the extracellular secretion of CD80, CD206, TNFα, IL-10 and CCL2 was evaluated, as suggested previously^38^.

**Fig. 1:**
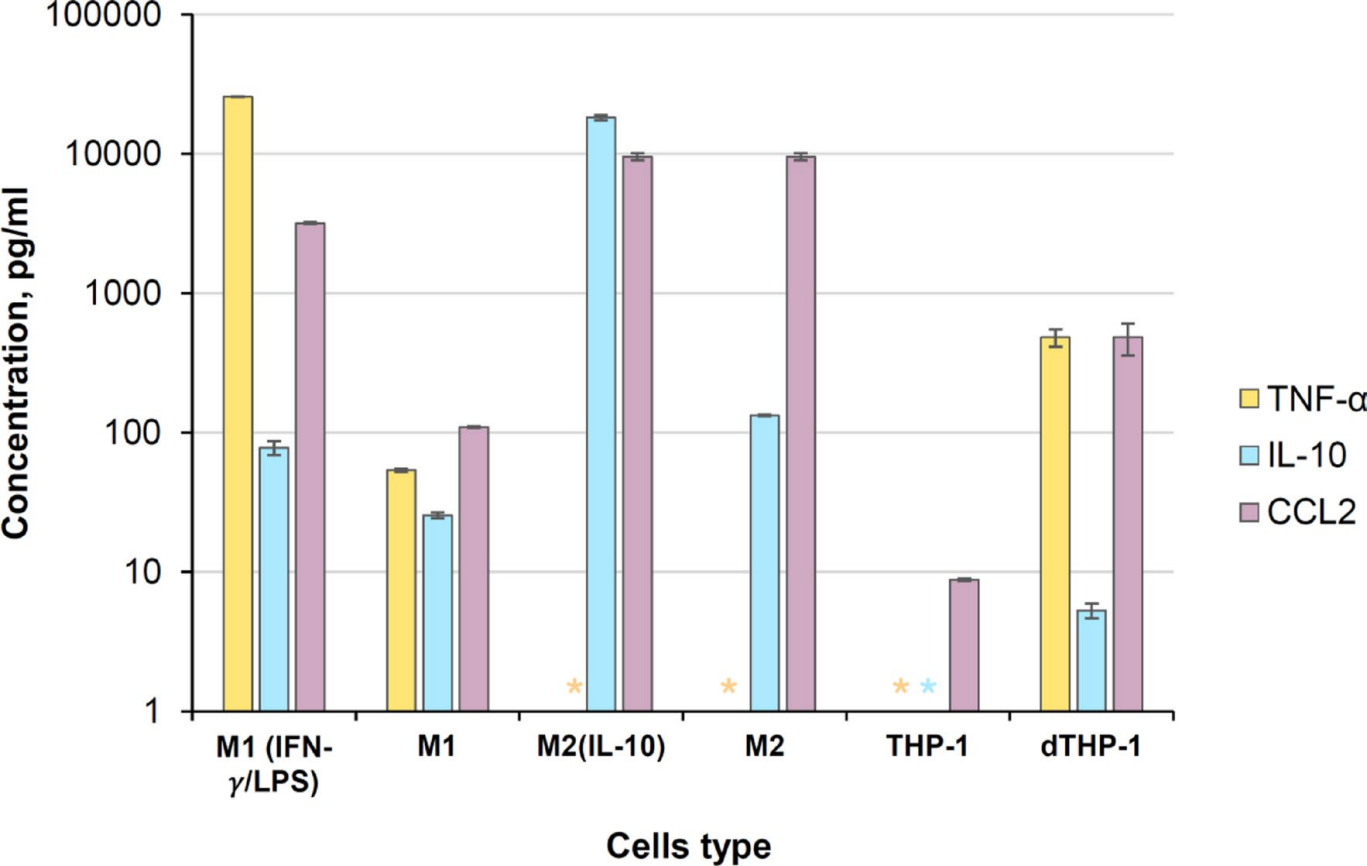
Extracellular levels of TNF-α, IL-10 and CCL2 from macrophage-like cells with different phenotypes. The symbol * stands for “not detected” (i.e., below the limit of detection). The data are presented as the means ± SDs (*n* = 3 independent experiments).

The data show the successful differentiation of human peripheral blood mononuclear cells into macrophages, resulting in phenotypes similar to those of M1-like and M2-like macrophages. M1-like macrophages express high levels of TNF-α (Fig. 1) and the proinflammatory marker CD80 (see SI Fig. S1), which is characterized by the classically activated macrophage phenotype. M1-like macrophages were successfully activated by IFN-γ and LPS, as demonstrated by the higher level of TNF-α in M1 macrophages (IFN-γ/LPS) than in M1 macrophages, which also confirms their proinflammatory phenotype (Fig. 1)^25^. Compared with M1-like macrophages, the successful differentiation of M2-like macrophages is confirmed by increased IL-10 and decreased TNF-α extracellular secretion, thus negatively regulating the secretion of proinflammatory cytokines^25^. M2-like macrophages are alternatively activated macrophages that are able to express a lower amount of the CD80 surface marker together with higher levels of CD206 (see SI, Fig. S1), which is in agreement with a previous report^39^. It has been reported that the high CCL2 level in human peripheral blood cells is correlated with an increase in CD206, a decrease in TNF-α and high extracellular secretion of IL-10, which promotes the M2-like phenotype in macrophages^40^, in agreement with the obtained results. Gschwandtner et al^41^. reported that CCL2 extracellular secretion can be stimulated by many different inflammatory triggers, including M-CSF, GM-CSF and TNF-α. This could explain the high level of CCL2 in M1-like cells and its increase after IFN-γ/LPS activation. For THP-1 macrophages, increased levels of TNF-α, CCL2 and IL-10 were observed after PMA treatment, confirming that differentiation occurred (Fig. 1). However, the CD80 level (see SI, Fig S1) was much lower than that observed for M1-like macrophages, in agreement with previous studies^42^. Additionally, recently, an increase in CCL2 in dTHP-1 macrophages was shown to promote polarization to the M1-like phenotype^43^, as observed in our data (Fig. 1).

Therefore, the results of the investigation of several surface markers and cytokine extracellular secretions suggest that the macrophages obtained from human peripheral blood cells correspond to the M1-like and M2-like phenotypes and that the dTHP-1 monocytes partially resemble proinflammatory macrophages.

#### Mitochondrial activity and proinflammatory effects of MSPs on different macrophage-like cells

**Fig. 2:**
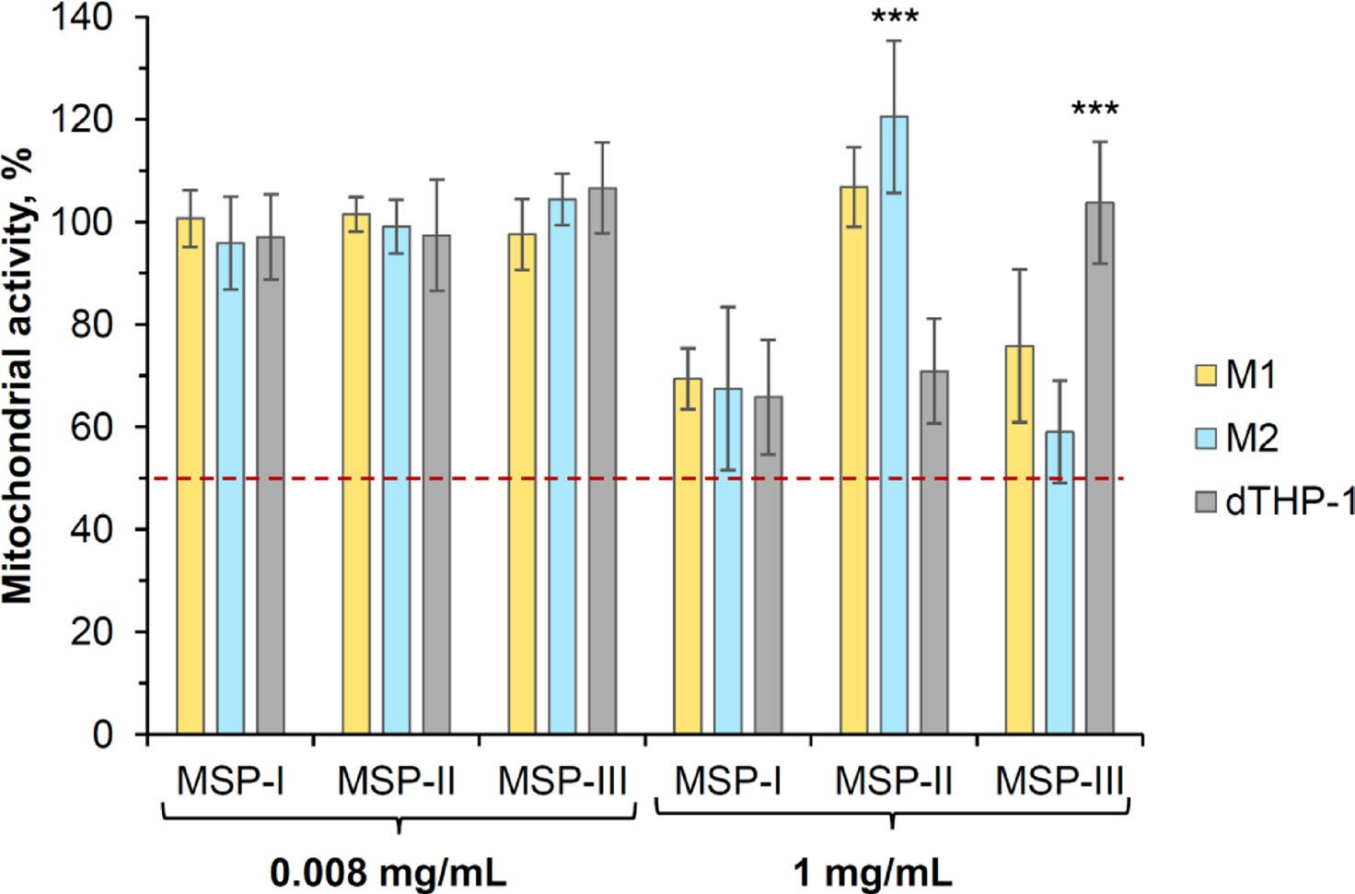
Mitochondrial activity after MSP exposure in different models of macrophage-like cells. MSPs were suspended in medium (in the presence of 5% FBS) at lower (0.008 mg/mL) and higher (1 mg/mL) concentrations and incubated for 4 h with the cells. Data are presented as the mean percentage of mitochondrial activity normalized to untreated cells ± SD from three independent experiments (*n* = 8 for each experiment). A significant increase was observed in mitochondrial activity after MSP-II exposure for M2-like macrophages and MSP-III exposure for dTHP-1 macrophages, and is indicated as ***p < 0.001 determined by one-way ANOVA with Tukey’s multiple comparison post hoc test.

The MTT assay was used to evaluate the mitochondrial activity of the macrophage-like cells after incubation with the three types of MSPs (Fig. 2). According to the OECD guidelines^26^, the MTT test is one of the most validated assays for evaluating the impact of new chemicals on cell viability.

The results revealed that more than 50% of the mitochondrial activity (i.e., greater than the IC_50_) was maintained after 4 h of incubation, irrespective of the particle concentration or cell type (Fig. 2). At the lower particle concentration (0.008 mg/mL), the mitochondrial activity was approximately 100% ± 5%. However, at higher particle concentrations (1 mg/mL), the mitochondrial activity of M1-like and M2-like macrophages decreased only after incubation with MSP-I and MSP-III (i.e., 69.4, 75.8% and 67.5, 59%, respectively) and not with MSP-II. Moreover, the mitochondrial activity of dTHP-1 macrophages significantly decreased only after incubation with MSP-I and MSP-II (approximately 65–70%), but it remained unchanged in the case of MSP-III. In terms of macrophage model dependence, MSP-I at 1 mg/mL had similar effects on the mitochondrial activity of all three cell types. However, 1 mg/mL MSP-II induced a significant decrease in mitochondrial activity only in dTHP-1 macrophages (i.e., 70.9%), with a significant increase in mitochondrial activity in M2-like macrophages (ANOVA, Tukey’s test, *p* ≤ 0.001). In contrast, MSP-III significantly decreased the mitochondrial activity in primary cells (76% for M1 and 59% for M2) but had no effect on dTHP-1 (ANOVA, Tukey test *p* ≤ 0.001).

Therefore, despite the same size of MSPs at a concentration of 1 mg/mL, they had different effects on macrophages, which can be explained by differences in the size and volume of pores, surface area, and dissolution rate, which led to different degrees of interaction with macrophages.

Primary M1- and M2-like macrophages were generally better tolerated by incubation with MSP-II, which in characteristics occupies an intermediate position between MSP-I and MSP-III, while dTHP-1 showed a significant decrease in mitochondrial activity during incubation with MSP-II, which may indicate that different macrophage models have different sensitivities to different combinations of particle structural properties.To understand the effect of MSPs on the local immunological response of macrophages, the secretion of the proinflammatory cytokineTNF-α was measured. Table 2 shows that MSPs did not induce TNF-α production in any of the types of macrophages tested in comparison with IFN-γ/LPS stimulation, which was used as a positive control.

**Table 2:** Release of the proinflammatory cytokine TNF-α from different types of macrophages after 4 h of incubation with MSPs (0.25 mg/mL dissolved in PBS, pH 7.4, in the presence of 5% FBS). IFN-γ/LPS stimulation was used as positive control. N.D. stands for “not detected” (i.e., below the limit of detection). The data are expressed in pg/mL as the means from 3 independent experiments ± SDs (*n* = 3 for each experiment).

dTHP-1	MSP-I	MSP-II	MSP-III	IFN-γ/LPS
	*N*.D.	*N*.D.	*N*.D.	2484.56 ± 44.55
M1	N.D.	N.D.	N.D.	7810.85 ± 94.08
M2	N.D.	N.D.	N.D.	6168.93 ± 76.72

The absence of TNF-α production after incubation with MSPs suggests that these particles may not induce a proinflammatory response in cells under the conditions tested. This could be explained by the size of the MSPs used (i.e., 2.2 μm). It has been shown^44^ that smaller MSPs (50–70 nm) may exhibit greater inflammatory potential in dTHP-1 cells, most likely due to their increased surface area and the possibility of greater interaction with cellular components. However, larger MSPs (100–1000 nm) may induce a softer or complete absence immune response, suggesting that a particular endocytosis mode, exclusively employed for the uptake of MSPs within a specific size range, is accountable for their size-dependent proinflammatory impact.

Importantly, the mitochondrial activity of cells, but not the proinflammatory effect, exhibited variability among different macrophage types upon exposure to various MSPs. While this finding demonstrates the importance of using different cell types for *in vitro* analysis, to ensure consistency and practicality, further experiments were conducted using dTHP-1 cells. This cell line is widely used as a standard macrophage model, provide higher reproducibility, and eliminate variability associated with donor differences, allowing us to focus on the influence of the physicochemical characteristics of MSPs (dissolution rate, surface properties) and dose.

### Dose-dependent effects of MSPs on mitochondrial activity

To assess the clinical risk and therapeutic window of new medicines, important values such as the ´no observed adverse effect level’ (NOAEL^14^, the highest dose without any observed toxic or adverse effects), the ‘low observed adverse effect level’ (LOAEL, the lowest dose with an observed toxic or adverse effect) and the half maximal toxic concentration (IC_50_) are typically evaluated in animal studies. Previously, Gies et al. reported a connection between the MTT assay in macrophages and NOAEL/LOAEL levels^45^. Similarly, the dose‒response curve of the MSPs was investigated via a mitochondrial activity test (i.e., MTT) as a hallmark of cytotoxicity. In parallel, the OECD guidelines classify new compounds as UN GHS No Category (i.e., absence of response after short-term exposure) when the cell mitochondrial activity is higher than 70%^26^ and as UN GHS Category 2 (i.e., irritant chemicals) when the mitochondrial activity is hampered lower than 50%^46^. Therefore, herein, we defined the *in vitro* NOAEL as the highest MSPs concentration that does not substantially change the mitochondrial activity (i.e., it is not significantly different from that of the untreated control) and the *in vitro* LOAEL as the lowest MSP concentration that results in a mitochondrial activity ≤ 70%. The lowest MSPs concentration that causes mitochondrial activity ≤ 50% is referred to as the IC_50_.

**Fig. 3:**
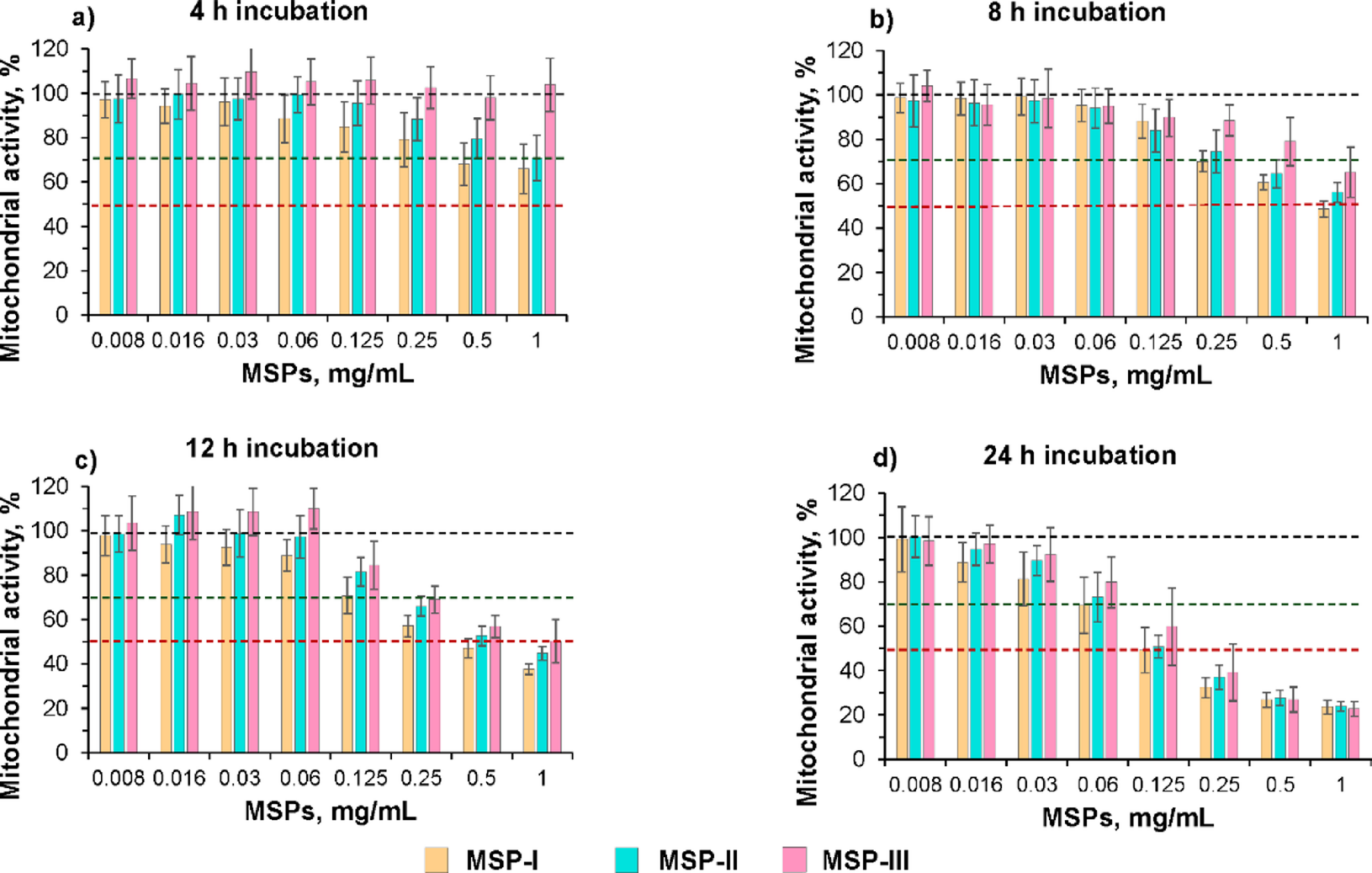
Dose‒response effects of MSPs on mitochondrial activity in cell medium supplemented with FBS. dTHP-1 cells were incubated with MSPs, and mitochondrial activity was assessed via the MTT assay at different incubation times (4, 8, 12, and 24 h). The incubation was performed in cell medium supplemented with 5% FBS, which is not a dissolving medium for MSPs. Data are presented as the mean percentage of mitochondrial activity normalized to untreated cells ± SD from three independent experiments (*n* = 8 for each experiment). The dotted lines at 100%, 70% and 50% mitochondrial activity define the NOAEL, LOAEL and IC_50,_ respectively.

One factor that plays an important role in particle toxicity is the medium in which cells and particles are placed during experiments^47^. Therefore, two types of liquids were used: RPMI 1640 (ATCC modification) in the presence of FBS, which is used for THP-1 culture to maintain cell functionality, and SLF, which resembles the lining fluid in the lungs^24^. In the first case, in the presence of FBS, the MSPs do not dissolve (i.e., no dissolving medium for the particles), whereas in the case of the SLF, the particles dissolve (i.e., dissolving medium for the particles) at the time rate presented in Table 1. For the cell medium sample, serial dilutions of MSPs were prepared in PBS to ensure reproducible particle dispersion under the experimental conditions and a final FBS concentration of 5%. To assess the role of particle dissolution, measurements were taken at three time points (4, 8, and 12 h), which correlated with twice the amount of time expected for 50% particle dissolution in SLF (Table 1) and an end point at 24 h. Figure 3 shows the mitochondrial activity after incubation with MSPs suspended in cell medium at different concentrations. After 4 h of incubation, the NOAEL and LOAEL values for both MSP-I and MSP-II were determined to be 0.03 mg/mL and 0.5 mg/mL, respectively (Fig. 3a). In contrast, MSP-III did not have any toxic effects after 4 h of incubation, regardless of the particle concentration. However, this could be a false negative due to the reduced sensitivity of dTHP-1 to MSP-III, as already discussed above (Fig. 2).

When the incubation time was increased to 8 h, a greater cytotoxic effect was observed (Fig. 3b); however, the mitochondrial activity remained above 70% at concentrations lower than 0.25 mg/mL (i.e., LOAEL) and approximately 100% at concentrations up to 0.06 mg/mL (i.e., NOAEL). When the incubation time was further increased to 12 h and 24 h, a strong dose-dependent cytotoxic effect was observed for all the particles (Fig. 3c, d). However, the mitochondrial activity was maintained at 70% or greater when the mixture was incubated for 12 h with 0.125 mg/mL MSPs (i.e., LOAEL), whereas no adverse effects were detected at concentrations up to 0.016 mg/mL (i.e., NOAEL). Finally, after 24 h of incubation, the cell mitochondrial activity remained above 70% for concentrations lower than 0.06 mg/mL (i.e., LOAEL), irrespective of the particle type.

**Fig. 4:**
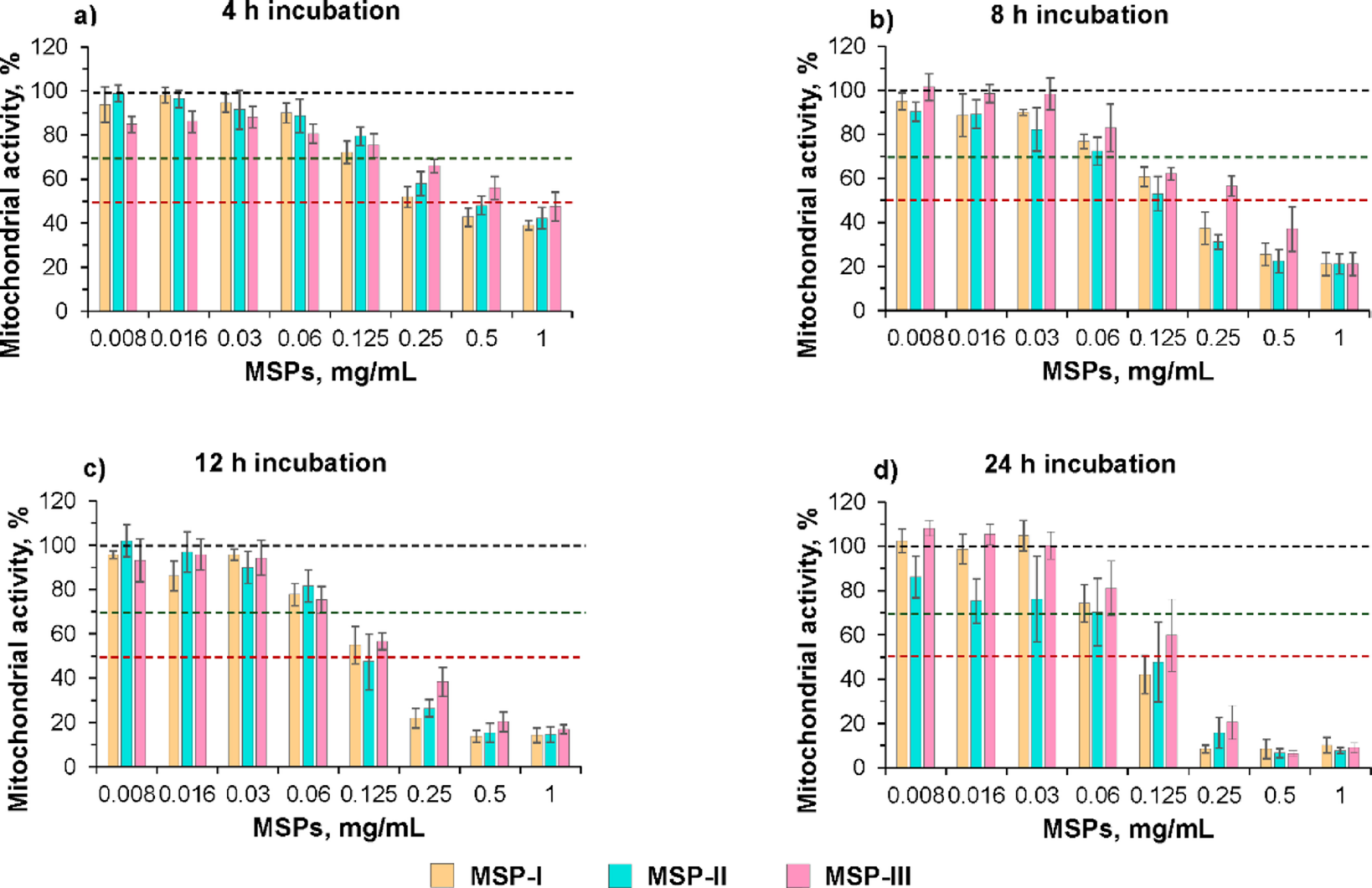
Dose‒response effects of MSPs on mitochondrial activity in SLF. dTHP-1 cells were incubated with MSPs, and mitochondrial activity was assessed via the MTT assay at different incubation times (4, 8, 12, and 24 h). The incubation was performed in SLF, which is a dissolving medium for the MSPs. Data are presented as the mean percentage of mitochondrial activity normalized to untreated cells ± SD from three independent experiments (*n* = 8 for each experiment). The dotted lines at 100%, 70% and 50% mitochondrial activity define the NOAEL, LOAEL and IC_50,_ respectively.

**Fig. 5:**
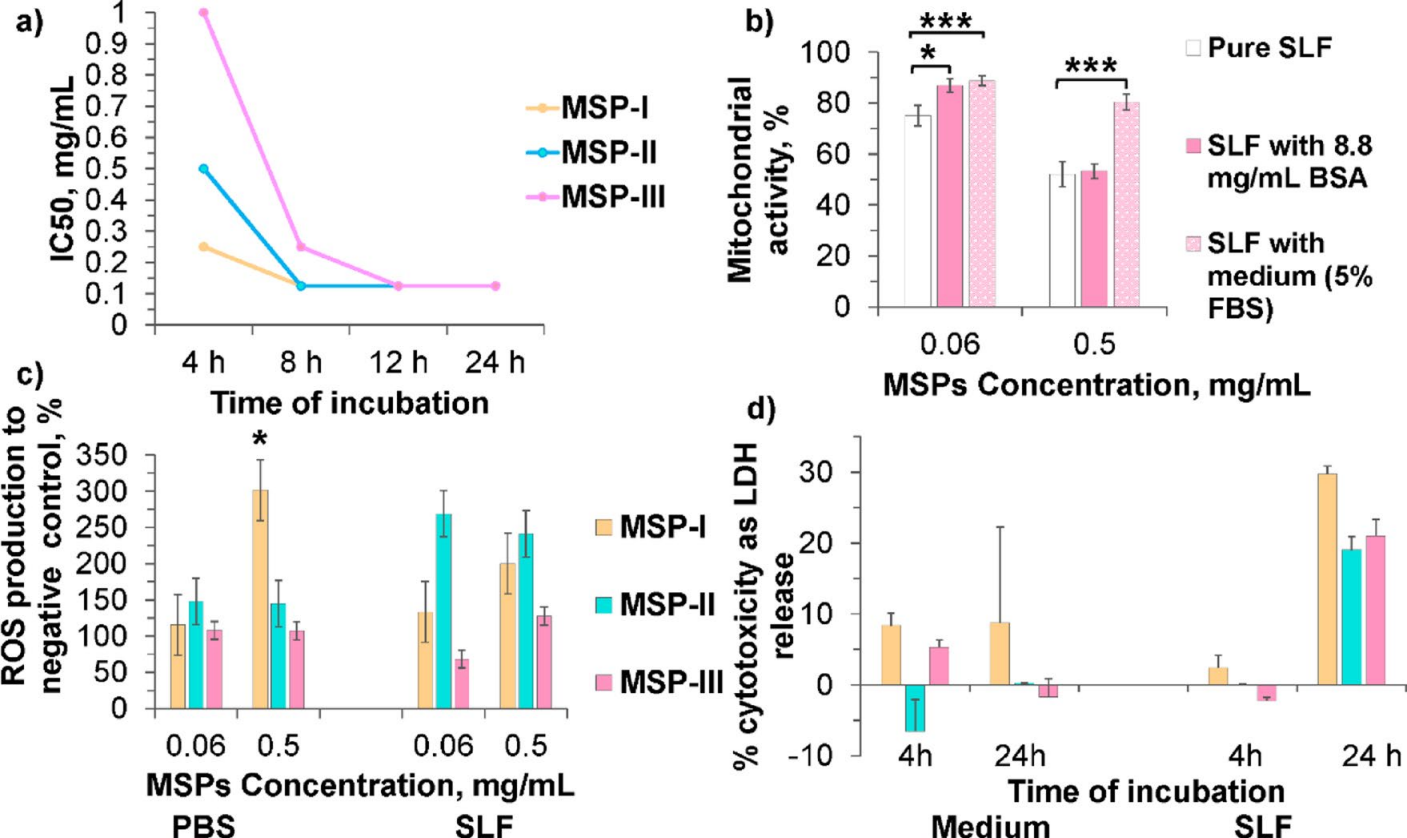
Relationship between particle dissolution and toxicity, analysis of oxidative stress and cell membrane integrity. **a**) Correlation between IC_50_ and incubation time in SLFs for all three MSPs. **b**) Influence of the ´protein corona´ on mitochondrial activity after 4 h incubation with MSP-III suspended in SLFs without or with 5% FBS or 8.8 mg/mL albumin (i.e., BSA). Significant difference is observed between MSP-III in pure SLF and MSP-III in SLF supplemented with FBS, regardless of the particle concentration (*p* < 0.001). At MSPs-III concentration 0.06 mg/mL, a protective effect is also evident in the presence of albumin (*p* = 0.018), whereas no protective effect is observed at 0.5 mg/mL. Data are presented as the mean ± SD from three independent experiments (*n* = 8 for each experiment). **c**) ROS production levels after 4 h depending on whether MSPs were suspended in PBS or SLF (*p* = 0.034). Significant difference is observed between MSP-I and MSP-III in PBS at particles concentration 0.5 mg/mL (*p* = 0.049). MSP‑II induces the highest ROS values in SLF after 4 h, matching its T50%, regardless of the particle concentration (*p* < 0.05). The data are presented as the means ± SEMs from three independent experiments (*n* = 4 for each experiment). **d**) LDH release after incubation with 0.06 mg/mL MSP at different incubation times, dissolution media and MSP types. The data are presented as the means ± SDs from three independent experiments (*n* = 3 for each experiment).

Figure 4 shows greater toxicity and stronger dose-dependent effect in SLF than those observed for the incubation tests in the cell medium. After 4 h of incubation with 0.125 mg/mL MSPs dissolved in SLF, the mitochondrial activity was 70% or greater (i.e., LOAEL) for all particle types, and no measurable difference was observed for concentrations up to 0.008 mg/mL (i.e., NOAEL) (Fig. 4а). These values are roughly four times lower than the values obtained when MSPs were suspended in cell medium (Fig. 3). When the incubation time was increased to 8 h, a greater cytotoxic effect was observed, and the LOAEL particle concentration decreased to 0.06 mg/mL (Fig. 4b). Interestingly, beyond 8 h, the LOAEL, NOAEL and IC_50_ values did not change as a function of the incubation time (Fig. 4b, c, d). According to the unloaded particle dissolution profiles in SLF (Table 1), after 8 h, both MSP-I and MSP-II were completely dissolved, and therefore, the toxicity remained unchanged. However, in a previous study, we showed that during dissolution in SLF, there is spontaneous formation of nanoparticles^5^ that are more toxic to macrophages than microparticles are^44^. In agreement with the data presented here, at concentrations higher than the LOAEL (i.e., 0.06 mg/mL), significantly lower mitochondrial activity was observed for all particle types in SLF than in the cell medium after 8 h of incubation (*p* < 0.001). To support this hypothesis, MSP-III, which has a longer dissolution time, appears to have a safer profile than MSP-I and MSP-II up to 8 h (i.e., IC_50_−4 h: 1 mg/mL vs. 0.5 mg/mL, IC_50_−8 h: 0.25 vs. 0.125 mg/mL, *p* < 0.001). Furthermore, after 12 h of incubation, when MSP-III is expected to be fully dissolved (T_50_= 6 h), the IC_50_ values are the same for all three types of particles. In fact, there was a strong correlation between the incubation time needed to reach the IC_50_ and the T_50_ of the particles (Fig. 5a), with the IC_50_ stabilizing around the dissolution time for each particle.

### Influences of the ‘protein corona’ on the cytotoxic effects of MSPs

Notably, the presence of FBS in the cell medium (Fig. 3) may affect the cellular uptake of nanoporous particles and the cytotoxic effect^28^. After entering pulmonary pathways, micro- and nanoparticles interact with various proteins in physiological fluids, such as lung fluid, and, by protein adsorption, form a ‘protein corona’ around the particles, which may affect the various functions of the particles, as well as their cytotoxicity^48^. This protein layer can influence particle recognition by the immune system, alter the response of macrophages, and directly affect the surface properties of the particles and their further biological fate^47^. Kuschnerus et al. elucidated the ‘hard’ and ‘soft’ coronas on different types of MPSs from serum proteins, which suggests specific protein adsorption profiles that depend on the shape of the particles and their proportion^48^. Our results in Figs. 3 and 4 show that the mitochondrial activity is much greater when the MSPs are suspended in the cell medium (i.e., not dissolving buffer) with 5% FBS than in SLF. Moreover, the independence of the effect on particle type after 24 h of incubation may be explained by the dissolution of the MSP (Fig. 5a) and/or by the formation of a ´protein corona´ under *in vitro *conditions, as reported previously for albumin^49^. To investigate the possibility of a “protein corona” in our experiments and understand the “protein corona” effect in the lung, further cytotoxicity tests were conducted in SLFs supplemented with FBS (5%) or albumin (8.8 mg/mL), the major protein constituent in the SLF formula^50^.

Figure 5b shows a significant difference in the mitochondrial activity between dTHP-1 cells exposed to MSPs suspended in pure SLF and those exposed to MSPs in SLF supplemented with FBS, regardless of the particle concentration (*p* < 0.001), which indicates the protective properties of the serum protein corona. In parallel, the protective effect of albumin was also observed when the MSP concentration was close to the LOAEL (i.e., 0.06 mg/mL, *p* = 0.018) but not when the concentration was increased to 0.5 mg/mL. Albumin is the most abundant protein present in FBS (19% of the total amount of protein)^51^, and it has been reported earlier that spherical MSPs form relatively homogeneous ‘soft’ and ‘hard’ protein coronas with relatively high albumin contents^48^. A caveat with these experiments is that the SLF was diluted with cell culture medium and therefore could have reduced the dissolution rate of the MSPs. These results fully agree with previous investigation showing as the protein corona formed in the presence of serum proteins may mediate biological and toxicological responses and thereby provide protective coverage and mitigate cytotoxicity^52^. The different effects of FBS and BSA on mitochondrial activity, depending on the concentration of MSPs (Fig. 5b), can probably be explained by the formation and composition of the protein corona on the MSPs surface. In pure SLF, where proteins are absent, MSP´s naked surface promotes direct interaction of particles with the cell membrane resulting in decreased cell metabolism. Conversely, the presence of FBS and BSA contributes to the formation of the protein corona reducing the direct stress caused by MSPs at lower concentrations and maintaining cellular activity^47^.

At higher concentrations of MSPs, the protective effect of the protein corona is likely to be insufficient, resulting in decreased mitochondrial activity of cells incubated in BSA-containing SLF, whereas RPMI medium with FBS, due to a more complex and functionally varied composition, still provides partial protection.

dTHP-1 cells were used as a macrophage model. For all experiments p values were calculated via ANOVA with Tukey’s multiple comparison post hoc analysis; Significance thresholds were defined as *p < 0.05, ***p < 0.001.

### ROS production could be related to endosomal rupture

The observed mitochondrial dysfunction could be generated as a cascade-response effect after MPSs internalization. MSPs generate ROS after their phagocytosis, leading to mitochondrial and inflammatory sequelae^30,53^. In human lung epithelial cells, ROS can cause permeabilization of the outer mitochondrial membrane, which releases soluble proteins (in particular cytochrome c, which binds to apoptosis protease activating factor-1) from the intermembrane space into the cytosol, where they are involved in the activation of caspase-3 and caspase-9^53^. Therefore, understanding the effects of nanocarriers on mitochondrial function and ROS generation is critical for assessing their safety and potential toxicity while new approaches for drug delivery are being developed. No MSP concentration dependency on ROS production was observed (see Supplementary Information, Fig. S2). It is possible that the concentration of MSPs is too high, and a plateau has been reached. However, at 0.06 mg/mL MPSs, negligible changes in ROS production (Fig. 5c) and mitochondrial activity (Fig. 3) were observed, whereas at higher concentrations (i.e., 0.5 mg/mL), high ROS production was inversely proportional to mitochondrial activity. Moreover, a statistically significant effect on the ROS level was observed depending on the medium in which the particles were suspended (*p* = 0.034), which was most likely due to the dissolution of the particles in the SLF. Compared with that in PBS (i.e., nondissolving medium), the production of ROS in SLF (i.e., dissolving medium) drastically increased. Interestingly, MSP-II also resulted in maximum ROS production in SLF after 4 h of incubation, which was equivalent to its T50% (*p* < 0.05). It is likely that at T50%, there is a high concentration of nanoparticles in the medium, as shown in our previous study^5^, drastically increasing ROS production. An increase in ROS production has also been linked to the formation of nanoparticles in other previous reports^29,54^, which is extremely important for drug delivery, since an increase in ROS production has been linked to endosomal rupture, increasing the efficiency of vaccine-mediated delivery of intracellular antigens^54^. Thus, the results indicate the influence of ROS on the mitochondrial activity of cells, albeit only in the case of high concentrations of particles, and highlight the importance of controlling the particle dissolution time for safe use.

### Cellular damage is linked to mitochondrial impairment

Importantly, the MTT assay revealed a dose-dependent effect of MSPs on macrophage-like cells, and the ROS data correlated with mitochondrial impairment and the production of ROS. To understand whether the observed impairment of cell metabolism caused by MSPs eventually leads to cell death, an additional LDH release test, a widely used indicator of cytotoxicity or cellular damage, was performed^55^. LDH is an enzyme located in the cytosol, but when the cell membrane is damaged or when cells die, LDH is released into the medium^56^. As expected, the LDH release assay (see SI, Fig S3) confirmed a dose-dependent effect when the particles were suspended in cell medium (containing 5% FBS), which was in agreement with the MTT results (Fig. 3). However, no clear, strictly dose-dependent effect was observed when MSPs was dissolved in pure SLF (see the SI, Fig. S3). At a particle concentration of 0.06 mg/mL in cell medium (in the presence of 5% FBS), the low LDH release after 4 h and 24 h (Fig. 5d) agreed with the MTT data (Fig. 3), as no LDH release or low LDH release was observed. Moreover, after 4 h of incubation with 0.06 mg/mL MSPs in SLFs, LDH release was negligible, which was in agreement with the observed mitochondrial impairment (Fig. 4a) but inconsistent with the observed ROS production (Fig. 5c), where a peak was observed for MSP-II in connection with its half-time dissolution particle rates. Although MSP-II induces early increases in ROS levels, the lack of increases in LDH levels indicates the absence of any signs demonstrating functional cell damage associated with LDH release.

After 24 h of incubation in SLF, a greater level of LDH was observed (i.e., approximately 20–30%, Fig. 5d), which is in strong agreement with the MTT results (i.e., approximately 70% mitochondrial activity, Fig. 4d). Moreover, a statistically significant difference in LDH release and particle type, as well as incubation time, was detected when the particles were suspended in the SLF (Fig. 4d), which is in full agreement with the ROS results and the half-time dissolution particle rates (T50%) in the SLF (i.e., MSP-I * *p* < 0.05). Notably, after both 4 h and 24 h of incubation, the LDH release of MSP-II suspended in medium (containing 5% FBS) was either very low or negative (i.e., lower than the spontaneous LDH release). The negative LDH values observed for such low concentrations of MPS can be explained by the ability of the particles to inactivate or absorb LDH^57,58^, which is confirmed directly by our present results (see the SI, Fig. S4). Moreover, very strong negative release of LDH was detected at a concentration of 1 mg/mL. The shorter half-dissolution time in the SLF (T50% = 2 h, Table 1) likely led to an increased surface area and, subsequently, an increase in the amount of LDH absorbed.

Our results indicate that the observed decrease in LDH levels is unlikely to reflect a genuine reduction in cytotoxicity. Instead, it is most probably a consequence of the interaction between MSPs and the enzyme, leading to LDH adsorption or inactivation. This suggests that the LDH assay may be an unreliable standalone indicator of cytotoxicity for certain nanomaterials.

At the same time, the obtained data have analytical value, as they demonstrate the susceptibility of the LDH assay to the structural properties of mesoporous particles. These findings broaden the current understanding of MSPs–protein interactions *in vitro* and show that such interactions can influence LDH measurements, which is an important consideration for the correct interpretation of cytotoxicity data in nanomaterial research.

Although our data indicate that MSPs can interact with LDH, potentially leading to adsorption or partial enzyme inactivation and thereby reducing assay reliability, the measured LDH levels in dTHP‑1 cells remained consistently low or even negative when MSPs were suspended in medium containing 5% FBS and incubated with the cells for both 4 h and 24 h. This suggests that, despite the methodological limitations, LDH release did not exceed the expected baseline and did not indicate membrane damage under these experimental conditions. Therefore, while the LDH assay should not be interpreted as a standalone measure of cytotoxicity for MSPs, the overall LDH profile supports the conclusion that MSPs exposure under these conditions does not induce detectable cytotoxic effects in THP‑1 cells, the observed toxic effects (i.e., both cell membrane disruption and mitochondrial activity) on macrophages at low concentrations (i.e., up to 0.06 mg/mL) are low. Some differences in the results of the MTT and LDH analyses point to the involvement of complicated mechanisms in the interaction between MSPs, macrophages and proteins. To elucidate and understand these mechanisms further, additional interdisciplinary studies encompassing biological, physical, and chemical perspectives are necessary.

### Lack of lysosomal membrane permeabilization at non‑toxic MSPs concentrations

**Fig. 6:**
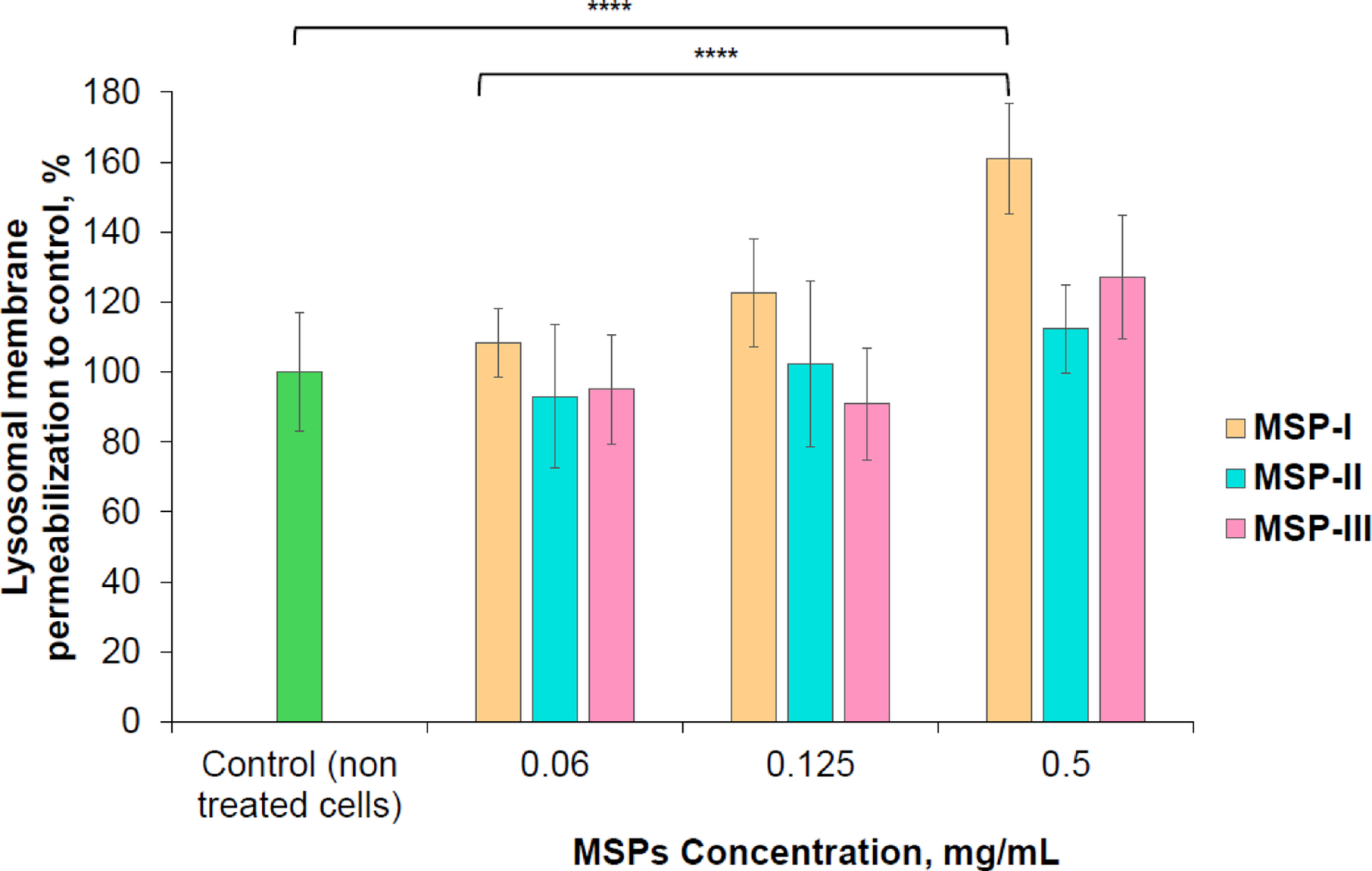
Dose‑dependent lysosomal membrane permeabilization in dTHP‑1cCells treated with MSPs.

Since the increase of ROS is a well-described factor capable of disrupting lysosomal membrane stability and causing LMP, an evaluation of the potential link between ROS generation and changes in lysosomal stability was performed using the LMP method after 4 h incubation dTHP-1 cells with three types of MSPs (see Fig. 6).The obtained results demonstrate a clear correspondence between ROS dynamics and LMP levels in cells exposed to MSP-I. Concentrations of 0.06 and 0.125 mg/mL did not cause statistically significant deviations from the control, indicating preserved lysosomal integrity under conditions of low particle burden. In contrast, increasing the concentration to 0.5 mg/mL resulted in a statistically significant concentration‑dependent increase in LMP (*p* < 0.0001). The higher porosity, larger specific surface area, and faster dissolution rate of MSP-I likely facilitated more pronounced interactions with intracellular compartments, resulting in a concentration dependent destabilization of lysosomal membranes (*p* < 0.0001). This effect fully aligned with the ROS profile, as 0.5 mg/mL MSP-I induced the most pronounced ROS production, whereas ROS levels at 0.06 mg/mL did not differ from the control (see Fig. 5c).

Thus, lysosomal membrane disruption occurred only under conditions associated with elevated oxidative stress, which is consistent with the mechanistic concept of ROS‑associated lysosomal stress. These observations suggest that oxidative imbalance may represent one of the key early events that precedes or contributes to the loss of lysosomal integrity upon exposure to high concentrations of MSP-I^59^.

In the case of MSP-II and MSP-III, no changes in LMP were detected at any tested concentration, which indicates that particles with lower pore volume, smaller surface area, and slower dissolution did not induce detectable lysosomal membrane disruption, and is fully consistent with the absence of ROS production in cells incubated with these MSPs. This concordance between two independent parameters (ROS and LMP) indicates that lysosomal alterations are not a universal property of MSPs but rather a specific cellular response to MSP-I under high‑dose conditions.

Overall, the data provide a coherent picture in which MSP-I induces concentration‑dependent effects involving oxidative stress and associated lysosomal destabilization. Lower concentrations of MSP-I did not exert significant effects on any of the evaluated parameters, while MSP-II and MSP-III remained biologically inert across all investigated endpoints. Although additional molecular markers such as caspase activation or assessment of autophagy‑related responses could further refine the mechanistic interpretation, however the present findings indicate that the studied MSPs exhibit a stable and predictable biological profile: low concentrations do not affect lysosomal integrity, whereas changes observed only at the high concentration display a clear ROS‑dependent pattern consistent with cellular adaptation mechanisms to excessive particle load^60^.

Lysosomal membrane permeabilization in dTHP‑1 cells after 4‑h exposure to three MSPs types with varying structural properties. MSP‑II and MSP‑III did not induce significant LMP at any tested concentration. In contrast, MSP‑I caused a concentration‑dependent increase in LMP (*p* < 0.0001), consistent with its higher pore volume, surface area, and faster dissolution rate. Data are presented as the normalized to non treated control means ± SD from two independent experiments (*n* = 6 for each experiment). p values were calculated via two-way ANOVA with Tukey’s multiple comparison post hoc analysis; Significance thresholds were defined as ****p < 0.0001.

While the present study focuses on macrophage responses to isolate the role of MSP dissolution kinetics on intracellular toxicity and inflammatory outcomes, earlier work addressed complementary aspects of pulmonary delivery at the lung barrier level. Specifically, phospholipid-coated MSPs were shown to interact with lipid lung surfactant (e.g. DPPC), penetrate bronchial mucus, and enhance epithelial tissue access and drug dissolution under airway-relevant conditions^61^. In contrast, the current study elucidates how dissolution-driven nanoparticle formation influences macrophage response, oxidative stress, and mitochondrial function. Future studies could integrate epithelial-macrophage co-culture and air-liquid interface models to bridge extracellular barrier interactions with immune cell-mediated responses.

### Drug release as a function of particle type

Our previous report highlighted the development of a dry powder formulation of MSP encapsulating clofazimine (MSP-CLZ) for tuberculosis treatment via pulmonary delivery. This formulation (i.e., MSP-III with a CLZ loading of approximately 10% w/w) was demonstrated to achieve 50% lung deposition (respirable fraction) and 16-fold greater drug dissolution than the pure drug, as well as killing efficacy for intra- and extracellular mycobacteria^5^. In the present study, investigating drug dissolution properties serves as a way of understanding the potential of using faster dissolving MSPs (i.e., MSP-I) instead of MSP-III. Figure 6a shows the release profile of CLZ from MSP-I and MSP-III in SLFs (SLF + DPPC), which initially contained CLZ percentages in CLZ-MSPs of 3.3 and 3.7% w/w for I and III, respectively. The dissolution kinetics of MSP-I reached a maximum drug release after 5 min (68%), which was greater than that of MSP-III (59%) (*p* = 0.007). MSP-III exhibited a maximum release of 60% at 10 min. In both cases, a decrease in concentration occurred, which was previously reported in our study, following the known “spring and parachute” model for supersaturating drug delivery systems, whereby rapid drug release generates a transient supersaturated state (“spring”), followed by a decline in concentration as the system relaxes toward equilibrium due to drug precipitation or crystallization (“parachute”)^62^. In particular, in both cases, the concentration of the drug released from MSP-I and MSP-III was above the minimum bactericidal concentration (MBC) against *M. tuberculosis*^63^ and above the solubility of CLZ in SLF + DPPC (reported previously^5^). For comparison, the intrinsic solubility of pure CLZ in SLF + DPPC, previously determined under identical experimental conditions^5^, is indicated as a reference level in Fig. 6a to contextualize the carrier-mediated enhancement in drug dissolution.

**Fig. 7:**
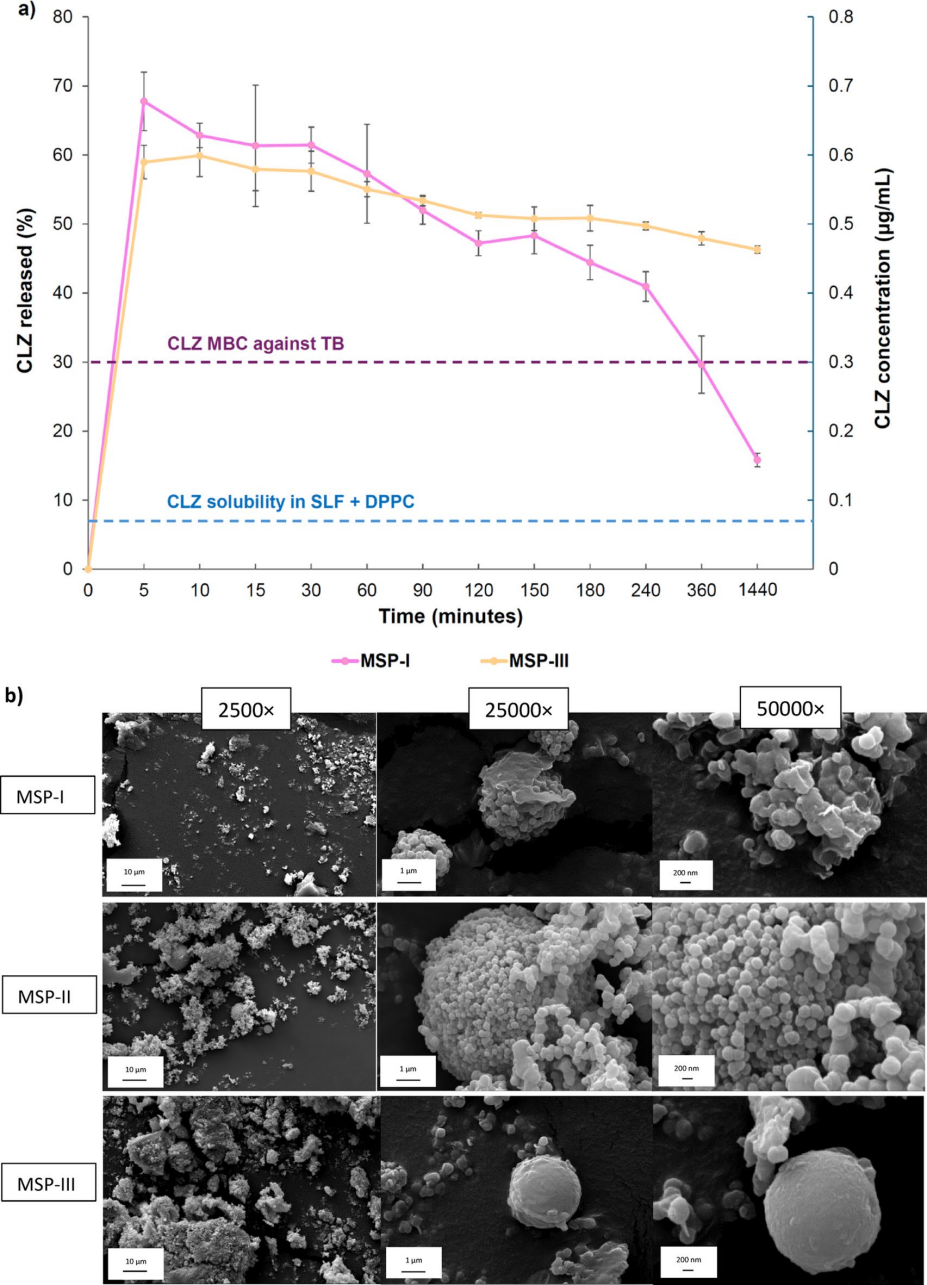
Drug release profile of CLZ-loaded MSPs and particle transformation of in simulated lung fluid. (**a**) Drug release profiles of CLZ–MSPs (30 mg/L) in SLF supplemented with DPPC using fast- and slow-dissolving carriers (MSP-I and MSP-III, respectively). The cumulative percentage of CLZ released (left y-axis) and the corresponding CLZ concentration (right y-axis) are shown over 6 h. Statistical analysis of released CLZ concentrations (ANOVA followed by Holm’s post hoc test) indicated a significant difference between MSP-I and MSP-III (*p* = 0.002). The dotted horizontal line represents the intrinsic solubility of pure CLZ in SLF + DPPC, previously determined under identical experimental conditions^5^. Data are presented as the mean ± SD from the independent experiment (*n* = 3 for the experiment). (**b**) Scanning electron microscopy images of MSP-I, MSP-II and MSP-III after a 24-h *in vitro* incubation in simulated lung fluid, illustrating distinct particle erosion and micro-to-nano transformation. Images were acquired at magnifications of 2500×, 25 000×, and 50 000×.

The enhanced apparent solubility and therefore achievement of a therapeutic concentration was previously attributed to MSPs^12^. Here, the greater drug release at short time points is likely related to the greater surface area (511 vs. 312 for MSP-I and MSP-III, respectively) rather than to the known different dissolution kinetics of the carrier in SLF. However, a rapid decline in dissolved CLZ concentration was observed for MSP-I after 4 h,, corresponding to the time point where complete dissolution of MSP I was expected, on the basis of a T50% of 2 h (Table 1), and is consistent with precipitation from a supersaturated state rather than directly measured precipitation. In contrast, MSP-III (T50% of 6 h) showed less pronounced precipitation, with more sustained CLZ dissolution kinetics after 4 h, resulting in a significantly higher concentration of CLZ for MSP-III than for MSP-I at 24 h (*p* < 0.001). Although MSP-III therefore exhibits superior stability and sustained CLZ release compared with MSP-I, Fig. 7 is not intended to rank formulations by drug dissolution performance. Instead, it illustrates how distinct carrier dissolution kinetics lead to different drug release behaviors, with rapid particle dissolution (MSP-I) promoting early drug supersaturation, whereas slower dissolution kinetics (MSP-III) support prolonged and more stable drug release. This result provides a mechanistic insight for designing MSPs according to different pulmonary therapeutic applications.

Figure 7b shows that MSP-I completely dissolved from the micro- to the nanoparticles within 24 h (aggregated). For MSP-II, a comparable micro- to nanoscale transformation was observed, characterized by the presence of aggregated nanosized primary particles, indicating intermediate erosion process. In contrast, for MSP-III, slight erosion of the micron-sized particles and remnants of the nanosized particles were observed, which was described as slower dissolution. It should be noted that the SEM-based erosion observations were performed on unloaded MSPs under identical SLF conditions and are therefore used here as representative indicators of carrier structural evolution during the release experiments with CLZ-loaded particles. In this study, MSP “dissolution” refers to the progressive structural erosion and micro-to-nano transformation of the amorphous silica particles in simulated lung fluid, as directly evidenced by SEM analysis (Fig. 7b), rather than a quantitative determination of silicon dissolution kinetics. Such kinetic analyses were previously established using dedicated methodologies in our earlier study^5,6^. Furthermore, to anchor these SEM observations in quantitative context, a previous work from one of the authors^19^ demonstrated a clear linear relationship between dissolution T50% and surface area across all tested particles. The surface area range of the tested MSPs varied from 304 to 558 m²/g, therefore including also the surface areas values of the three MSP types used in this study (312, 380, and 551 m²/g). Importantly, the T50% is calculated on the basis of a linear regression of dissolution vs. time up to 8 h; therefore, an extrapolation would suggest that complete dissolution of all MSPs would occur after 24 h^19^. The morphologies of the initial MSPs prior to dissolution are presented in Figure S5 for comparison. We hypothesized that after 4 h, the presence of micron-sized particles and aggregated nanosized particles in the case of MSP-III^5^ hinders the interactions between the released CLZ molecules; therefore, slower drug precipitation occurs (Fig. 7a).

Anwar et al^64^. conducted molecular dynamics simulations and concluded that large additives can completely inhibit crystal growth, whereas small additives only slow the process of nucleation. In this latter case, the particle can be part of the solute lattice. However, precipitation can also depend on many other factors, such as the strength of interaction of the small solute with the additive, the interfacial properties of each solute and the strength of self-association of the solute. Considering the case of CLZ-MSPs-III, the observed reduction in precipitation can thus be ascribed to the presence of micron-sized particles in the dissolved MSP-III, which likely inhibits CLZ precipitation.

In addition, from a therapeutic perspective, CLZ-MSP-I (3.3% w/w) and CLZ-MSP-II (3.7% w/w) reached maximum concentrations of 0.67 and 0.60 µg/mL, respectively. This concentration is almost half of the maximum concentration (1.25 µg/mL) reported before from an MSP-CLZ formulation^5^, where it was speculated that a single daily dose of 1.2–1.5 mg CLZ-MSP (9–10% w/w) delivering 1.25 µg/mL would effectively kill extracellular mycobacteria. This means that, from our latest experiments presented here, either the number of doses must be doubled, or a drug loading of 10% w/w is necessary. The latter approach would be more suited for ensuring that patients adhere to their medication regimen and minimizing the amount of excipient needed, which aligns with our overall approach of localizing the drug in the lungs without any adverse effects.

## Conclusions

This study presents an extensive investigation of the cytotoxic properties of three MSPs with different porosity properties and dissolution times using different models of macrophages. The mitochondrial activity was maintained above the IC_50_ in both primary human macrophages and dTPH-1 cells after 4 h of exposure to all the MSPs types at high and low concentrations (i.e., up to 1 mg/mL). The incubation with MSPs at a concentration of 0.06 mg/mL or less maintained the mitochondrial activity above 70% for up to 24 h (defined as the low observed adverse effect level ‘LOAEL’). When MSPs (i.e., SLFs) were dissolved in dissolving medium, the mitochondrial activity drastically decreased, suggesting that the nanoparticles formed from dissolved micron-sized silica particles^5^ had a more pronounced dose-dependent effect on macrophage viability than did the initial microparticles. To corroborate this hypothesis, the IC_50_ reached a plateau around the dissolution time for each particle, implying that particles with faster dissolution profiles are more toxic at short exposure times (i.e., MSP-I > -II > -III). However, at longer exposure times (i.e., 24 h), all the MSPs presented the same toxicity effect. We found that at the LOAEL level, the major protein present in the lung fluid, albumin, increased the mitochondrial activity, reaching the non-observed adverse effect level (i.e., NOAEL). Moreover, at the LOAEL, only a minor increase in ROS production was observed, whereas at higher concentrations (i.e., 0.5 mg/mL), high ROS production was observed, which can be linked to endosomal rupture, enabling potential intracellular antigen efficiency for vaccines. As a complement to the cytotoxicity test, the lysosomal membrane permeability study also showed a LOAEL level at MSPs concentration of 0.06 mg/mL. MSPs exposure did not induce TNF-α production in any type of macrophage, suggesting a weak immunological response in the macrophages.

Taken together, the results of these cellular studies suggest that MSPs at 0.06 mg/mL cause few or no adverse effects, regardless of the MSP type. In this case, the LOAEL dose of 0.06 mg/mL MSPs loaded with 10% w/w CLZ reached a therapeutic concentration close to the MIC_99_ of CLZ-MSPs (1.25 µg/mL) against mycobacteria. In terms of drug release kinetics, MSP-I resulted in a faster but less stable supersaturated solution in SLF, while MSP-III resulted in a more sustained dissolution profile than did MSP-I, confirming the demonstrated potential for more effective drug release. Further research will continue to optimize CLZ-MSPs for preclinical and clinical applications.

## Supplementary Information

Below is the link to the electronic supplementary material.

Supplementary Material 1

## Data Availability

All the data generated or analyzed during this study are included in this published article [and its supplementary information files].

## Abbreviations

AMs: Alveolar macrophages
AO: Acridine Orange
CLZ: Clofazimine
DPPC: Dipalmitoylphosphatidylcholine
LDH: Lactate dehydrogenase
LMP: Lysosomal membrane permeabilization
LOAEL: Lowest observed adverse effect level
MSPs: Mesoporous silica particles
MSP-CLZ: MSP encapsulating clofazimine
NOAEL: Nonobserved adverse effect level
ROS: Reactive oxygen species
SLF: Simulated lung fluid
