# Supplementary material for: Particle dissolution rate controls macrophage response and drug release from mesoporous silica inhalation carriers
Source: Sci Rep. 2026 Apr 1;16:11229. doi: 10.1038/s41598-026-46033-8 (PMC13046809; doi:10.1038/s41598-026-46033-8)
Supplement: Supplementary file 1 — Supplementary Material 1 [file 41598_2026_46033_MOESM1_ESM.docx]

**Particle dissolution rate controls macrophage response and drug release from mesoporous silica inhalation carriers**

Tetiana Yalovenko ^a, b^, Jesús E. Campos Pacheco ^a, b^, Gustav Sedelius ^a, b^, Emilie Schousboe ^a, b^, Antonia Alionte ^a, b^, Georgia Pilkington^с^, Anna Gustafsson ^a, b^, Sabrina Valetti ^a, b^

^a^ Biomedical Science, Faculty of Health and Society, Malmö University, 205 06 Malmö, Sweden;

^b^ Biofilms – Research Center for Biointerfaces (BRCB), Malmö University, 205 06 Malmö, Sweden;

^с^ Surface and Corrosion Science, KTH Royal Institute of Technology, SE-100 44 Stockholm, Sweden

Correspondence [*sabrina.valetti@mau.se](mailto:*sabrina.valetti@mau.se)

**Supplementary information**

**Content**

[Table S1. Simulated lung fluid compositions. 2](#_Toc139977098)

[Figure S1. Percentage of positive cells for the surface markers CD80 and CD206. 3](#_Toc139977099)

[Figure S2. Box plot depicting ROS production levels on dTHP-1 cells depending on MSPs concentration. 3](#_Toc139977100)

[Figure S3. LDH release after incubation dTHP-1 cells with MSPs](#_Toc139977101) 4

[Figure S4. Effect of MSP-II on LDH activity. 5](#_Toc139977102)

[Figure S5. Morphology of MSPs prior to dissolution 6](#_Toc139977103)

[Figure S6. Qualitative DLS intensity distributions of nano-sized particles formed during MSP-III dissolution in simulated lung fluid: PBS 1:1. 7](#_Toc139977103)

[**Method S1. Differentiation of human monocyte-derived macrophages 7**](#_Toc139977103)

[Method S2. Flow cytometry 8](#_Toc139977103)

[Method S3. Quantitative analysis of TNF-α, IL-10 and CCL-2 8](#_Toc139977103)

**Table S1.** SLF composition.

| Composition | Molarity (mM) |
| --- | --- |
| Magnesium chloride hexahydrate | 0.100 |
| Sodium chloride | 10.300 |
| Potassium chloride | 0.400 |
| Sodium sulfate anhydrous | 0.050 |
| Calcium chloride dihydrate | 0.250 |
| Sodium acetate | 1.161 |
| Sodium hydrogen carbonate | 3.100 |
| Sodium citrate dihydrate | 0.033 |

To prepare SLF + DPPC, 0.02 g of DPPC were added to 100 mL SLF, sonicated for 1 h at around 55 ºC and prepared according to [1].


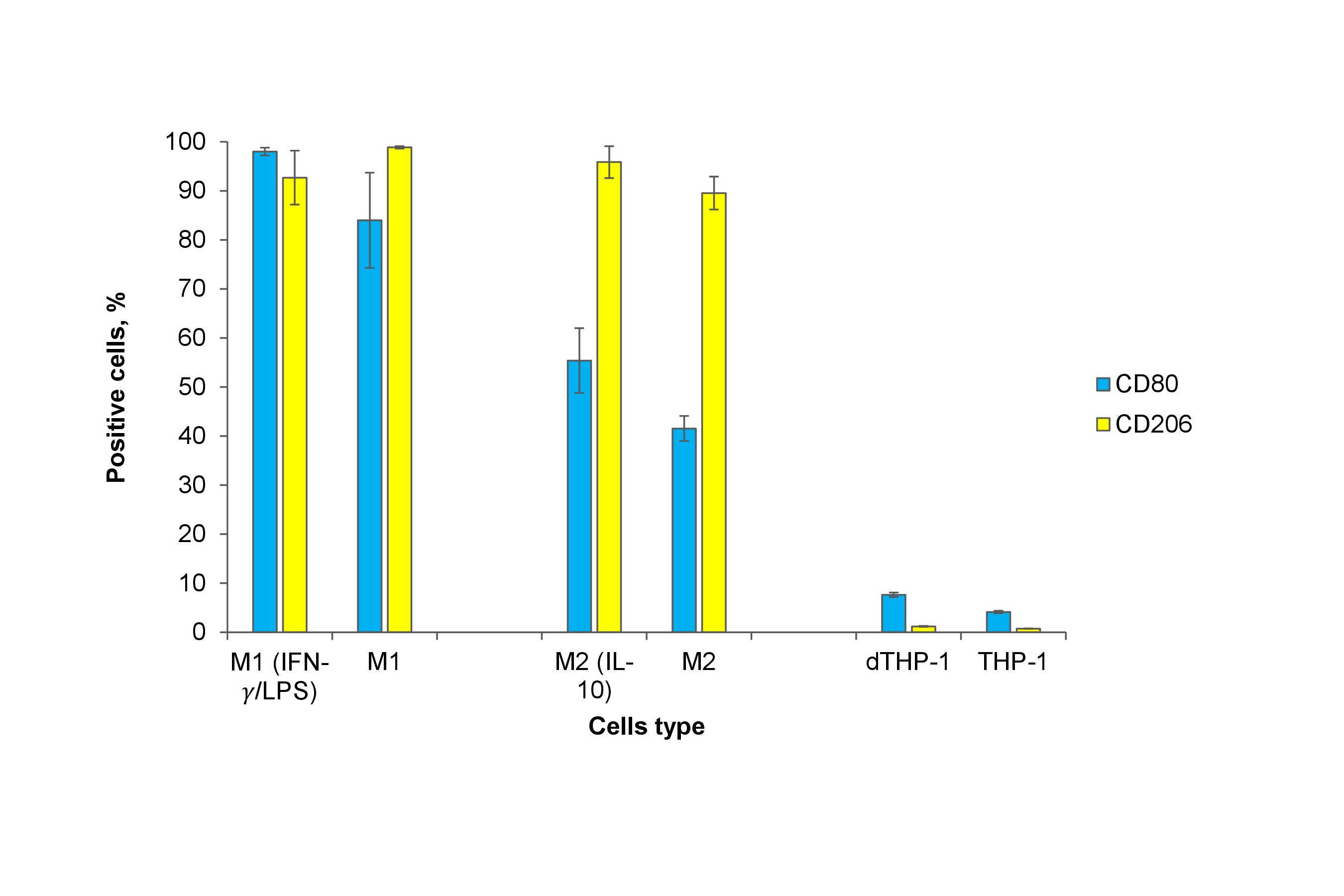
**Figure S1**: Percentage of positive cells for the surface markers CD80 and CD206 analysed by flow cytometry. Data are expressed as the portion of cells positive for the surface markers CD80 and CD206, with the isotype controls subtracted ± SDs (n=2 independent experiments).


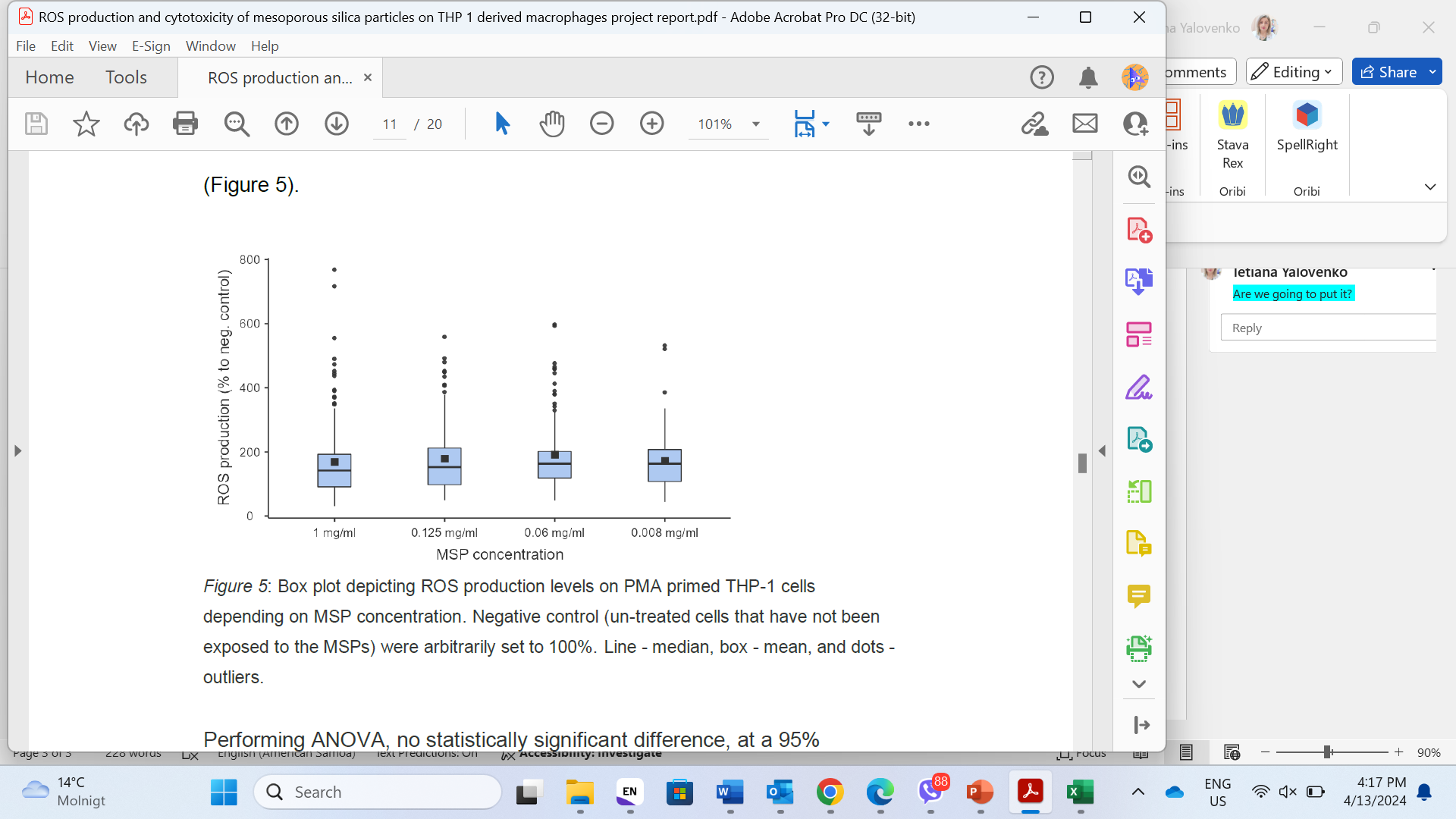


**Figure S2**: Box plot depicting ROS production levels on dTHP-1 cells depending on MSPs concentration. Negative control (un-treated cells that have not been exposed to the MSPs) were arbitrarily set to 100%. Line - median, box - mean, and dots - outliers.


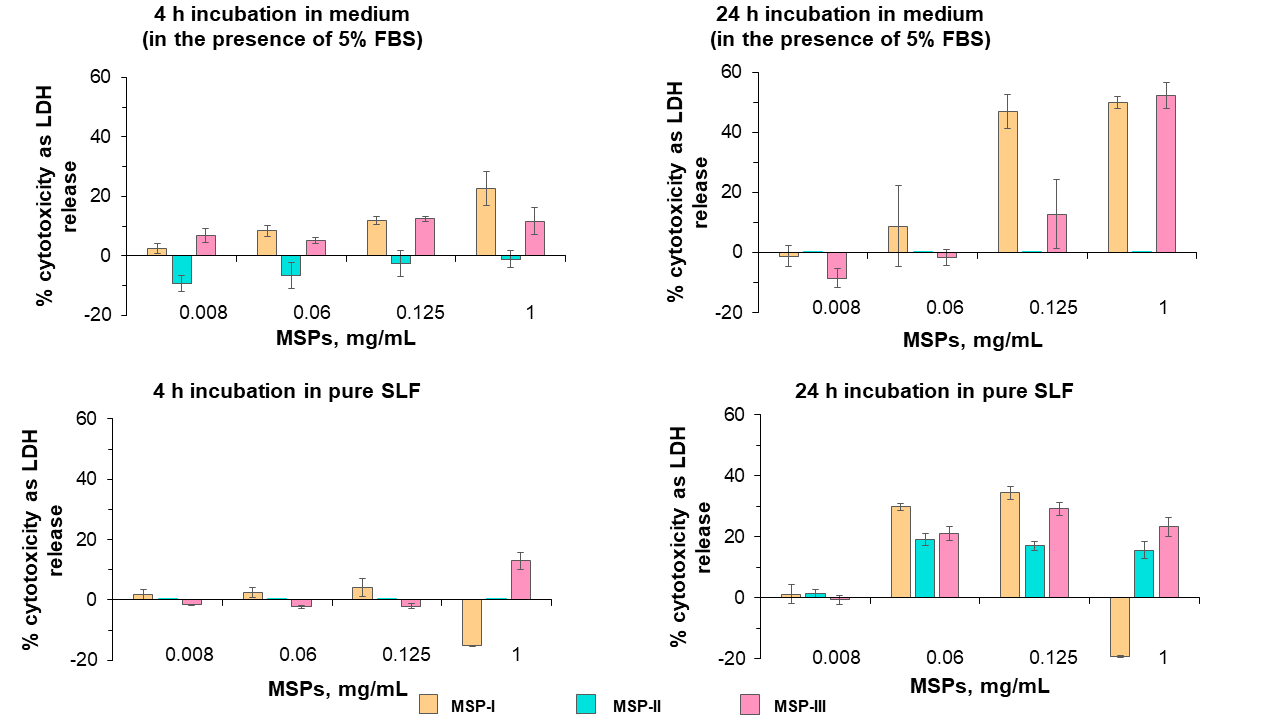


**Figure S3**: LDH release after incubation dTHP-1 cells with MSPs. LDH release from dTHP-1 cells was assessed following treatment with MSP-I, MSP-II, and MSP-III at particle concentrations of 0.008 mg/mL, 0.06 mg/mL, 0.125 mg/mL, 1mg/mL The particles were dissolved in PBS with medium (i.e., 5% FBS) (1:1) and pure SLF. The cells were subsequently incubated for durations for 4 h and 24 h and then assessed for LDH release in the culture supernatant. The data are presented as the mean ± SD for each concentration, with three replicates performed for each assay.


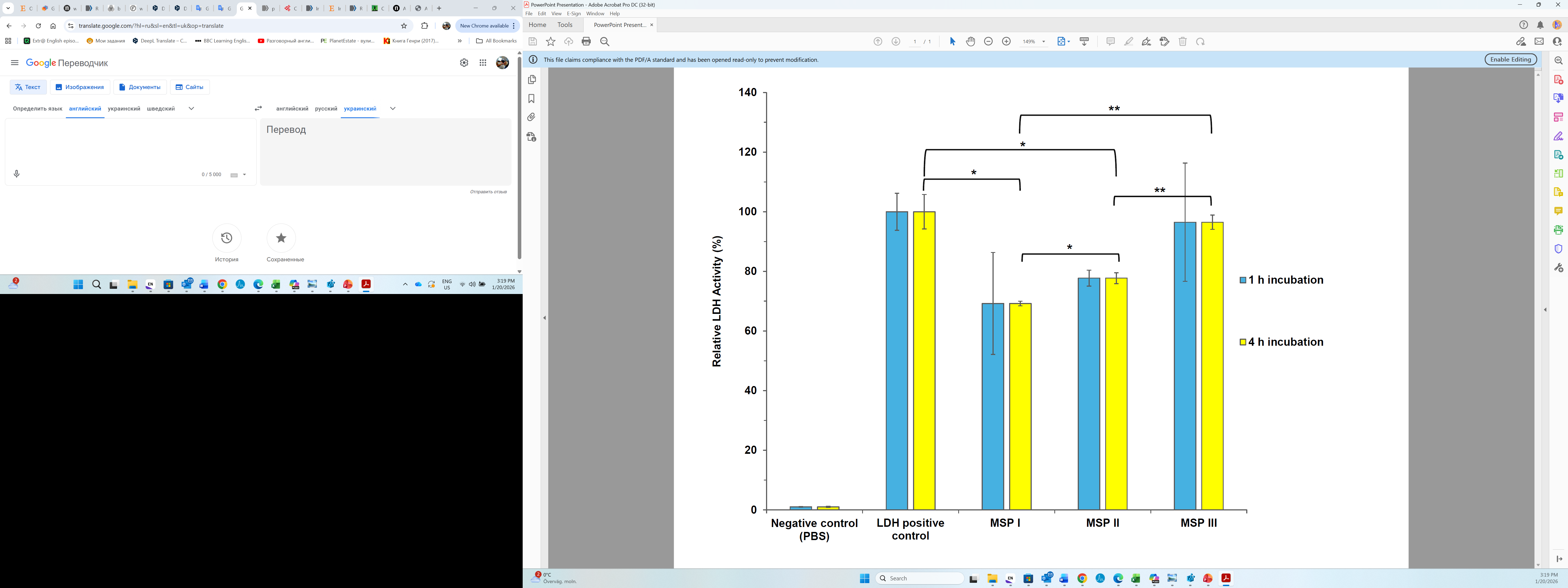


**Figure S4:** Effect of MSPs on LDH activity. LDH from the CyQUANT™ LDH Cytotoxicity Assay Kit (LDH Positive Control) was incubated for 1 and 4 hours in the presence and without 0.125 mg/mL MSPs. The presence of MSP-I and MSP-II significantly reduced LDH levels after 4 h of incubation, but not after 1 h. Comparison of MSPs revealed statistically significant differences in LDH activity at 4 h, demonstrating that the individual properties of MSPs (Table 1) affect LDH availability, including potential inactivation or adsorption.

The data are presented as the normalized to positive control means ± SDs (n=3 for each experiment). p values were calculated via ANOVA with Tukey’s multiple comparison post hoc analysis; Significance thresholds were defined as *p < 0.05, **p < 0.01.

**Figure S5:** The morphology of MSPs prior to dissolution was observed at magnifications of 2500×, 25000×, and 50000×.

**Figure S6:** Qualitative DLS intensity distributions of nano-sized particles formed during MSP-III dissolution in simulated lung fluid: PBS 1:1.

**Method S1. Differentiation of human monocyte-derived macrophages**

10–15 mL of donor’s blood was diluted with 50 mL PBS and layered over 15 mL Ficoll in two Falcon tubes (25 mL per tube). The samples were centrifuged at 400 × g for 40 min with minimal acceleration and deceleration to avoid mixing the layers. Buffy coats were collected and washed twice with 10 mL PBS. Flow cytometry confirmed that 25–30% of PBMCs were monocytes. The PBMCs were counted via the trypan blue exclusion test and subsequently seeded in 6-well culture plates (Thermo Scientific, USA) at a concentration of 1 × 10^7^ cells/well in 2 mL of monocyte attachment medium (MAM) and incubated for 1–5 h at 5% CO_2_ and 37°C. The MAM containing the nonadherent cells was aspirated, and the wells were washed three times with 1 mL of PBS. Two milliliters of cell culture medium containing either 50 ng/mL GM-CSF or 50 ng/mL M-CSF was added for M1 and M2 differentiation, respectively. After three days of incubation at 37°C and 5% CO_2_, 1 mL of cell culture medium was carefully removed from the top layer of each well, and the wells were supplemented with 1 mL of fresh medium containing double the concentration (100 ng/mL) of GM-CSF or M-CSF and incubated for an additional three days. For M1 activation, 50 ng/mL IFN-γ and 10 ng/mL LPS were added on Day 6 to each well, and for M2 activation, 10 ng/mL IL-10 was added, and the cells were incubated for 48 h. On Day 7, the culture supernatants were collected and stored in a freezer at -20°C for cytokine analysis (described in Section 2.9.). The cells were washed with PBS and then detached by adding 0.5 mL of Accutase per well, followed by incubation for 10 min at room temperature. The cell suspension was mixed well by pipetting. If detachment was not complete, a cell scraper was gently used to dislodge the cells. The suspension was centrifuged (Heraeus, Thermo Scientific, WA, USA) for 10 min at 400 × g and washed two times with 3 mL of PBS supplemented with 0.5% FBS. The washed cells were used for flow cytometry (described in Section 2.6.) A total of 10^4^ cells/well in RPMI were allowed to adhere for 24 h in 96-well plates before MSP treatment started.

**Method S2. Flow cytometry**

To evaluate the phenotype after THP-1 differentiation and human primary macrophage polarization via flow cytometry, 4 × 10^5^ THP-1 cells/mL were seeded into 6-well culture plates and differentiated as described in Section 2.4. The same conditions were followed for a control, with the exception of 50 ng/mL PMA for 48 h, which was used for the differentiation of this cell line. Human primary macrophages were prepared for flow cytometry as described in Section 2.5. Both cell lines were identically detached from the culture plates as described in Section 2.5. dTHP-1, M1, M2, M1 (IFN-γ/LPS), and M2 (IL-10) cells were counted via a flow cytometer. A disposable hemocytometer (C-chip, DHC-801) (NanoEnTek, Seoul, Korea) and Trypan blue were used to exclude dead cells. A total of 2 × 10^5^ cells were transferred to flow cytometry (FC) tubes and centrifuged at 300 × g for 5 min. The supernatants were completely aspirated, and the eluted cells were harvested as described and resuspended in PBS containing 0.5% FBS. They were then stained with APC-labeled anti-CD80 and PE-labeled anti-CD206, along with appropriately labeled isotype controls. Cell viability was assessed by staining with 7-AAD. The mean fluorescence intensities were collected between 585 and 540 nm for the PE fluorochrome, 670 nm longpass (LP) filter for the 7-AAD fluorochrome, and 675–625 nm longpass (LP) filter for the APC fluorochrome. Excitation was performed using the 488 nm line of an argon laser.

**Method S3. Quantitative analysis of TNF-α, IL-10 and CCL-2**

96–well microplates were coated with 100 μL/well of 4 μg/mL capture antibody, sealed, and incubated overnight at room temperature. Wells were washed with 400 μL of ELISA wash buffer using an automated washer and blocked with 300 μL of Reagent Diluent for at least 1 h before washing again.

Next, 100 µL of each sample was added according to the previously prepared plate layout, covered with an adhesive strip, and incubated for 2 h at room temperature. After washing, 100 μL of 50 ng/mL detection antibody was added and incubated for 2 h. Wells were washed again, followed by the addition of 100 μL of streptavidin‑HRP working dilution and incubation for 20 min at room temperature in the dark.

After a final wash, 100 μL of substrate solution was added, and the plates were incubated for 20 min in the dark before the reaction was stopped with 50 μL of stop solution. Absorbance was measured at 450 nm with background subtraction at 570 nm using a Tecan Safire microplate reader (Tecan, Switzerland).

**References:**

1. Son, Y.-J. and J.T. McConville, *Development of a standardized dissolution test method for inhaled pharmaceutical formulations.* International Journal of Pharmaceutics, 2009. **382**(1): p. 15-22.
